# The *Trypanosoma Brucei* KIFC1 Kinesin Ensures the Fast Antibody Clearance Required for Parasite Infectivity

**DOI:** 10.1016/j.isci.2020.101476

**Published:** 2020-08-20

**Authors:** Laurence Lecordier, Sophie Uzureau, Gilles Vanwalleghem, Magali Deleu, Jean-Marc Crowet, Paul Barry, Barry Moran, Paul Voorheis, Andra-Cristina Dumitru, Yoshiki Yamaryo-Botté, Marc Dieu, Patricia Tebabi, Benoit Vanhollebeke, Laurence Lins, Cyrille Y. Botté, David Alsteens, Yves Dufrêne, David Pérez-Morga, Derek P. Nolan, Etienne Pays

**Affiliations:** 1 Laboratory of Molecular Parasitology, IBMM, Université Libre de Bruxelles, 12, rue des professeurs Jeener et Brachet, 6041 Gosselies, Belgium; 2 Laboratory of Molecular Biophysics at Interface (LBMI), University of Liège-Gembloux Agro Bio Tech, 2, Passage des Déportés, 5030 Gembloux, Belgium; 3 School of Biochemistry and Immunology, Trinity College Dublin, Dublin 2, Ireland; 4 Louvain Institute of Biomolecular Science and Technology, Catholic University of Louvain, Croix du Sud 4-5, 1348 Louvain-la-Neuve, Belgium; 5 Institute for Advanced Biosciences, CNRS UMR5309, Université Grenoble Alpes, INSERM U1209, 38700 La Tronche, France; 6 MaSUN, Mass Spectrometry Facility, University of Namur, 61 Rue de Bruxelles, 5000 Namur, Belgium; 7 Laboratory of Neurovascular Signaling, Université Libre de Bruxelles, 12, Rue des Profs Jeener et Brachet, 6041 Gosselies, Belgium; 8 Center for Microscopy and Molecular Imaging (CMMI), Université Libre de Bruxelles, 12, Rue des Profs Jeener et Brachet, 6041 Gosselies, Belgium

**Keywords:** Immunology, Microbiology Parasite, Cell Biology

## Abstract

Human innate immunity to *Trypanosoma brucei* involves the trypanosome C-terminal kinesin *Tb*KIFC1, which transports internalized trypanolytic factor apolipoprotein L1 (APOL1) within the parasite. We show that *Tb*KIFC1 preferentially associates with cholesterol-containing membranes and is indispensable for mammalian infectivity. Knockdown of *Tb*KIFC1 did not affect trypanosome growth *in vitro* but rendered the parasites unable to infect mice unless antibody synthesis was compromised. Surface clearance of Variant Surface Glycoprotein (VSG)-antibody complexes was far slower in these cells, which were more susceptible to capture by macrophages. This phenotype was not due to defects in VSG expression or trafficking but to decreased VSG mobility in a less fluid, stiffer surface membrane. This change can be attributed to increased cholesterol level in the surface membrane in *Tb*KIFC1 knockdown cells. Clearance of surface-bound antibodies by *T. brucei* is therefore essential for infectivity and depends on high membrane fluidity maintained by the cholesterol-trafficking activity of *Tb*KIFC1.

## Highlights

• The *Tb*KIFC1 kinesin preferentially transports cholesterol-containing membranes
• *Tb*KIFC1 is absolutely required for parasite growth in mice
• Cholesterol trafficking by *Tb*KIFC1 confers high fluidity to the plasma membrane
• High membrane fluidity allows antibody clearance necessary for parasite infectivity

## Graphical Abstract

**Figure:**
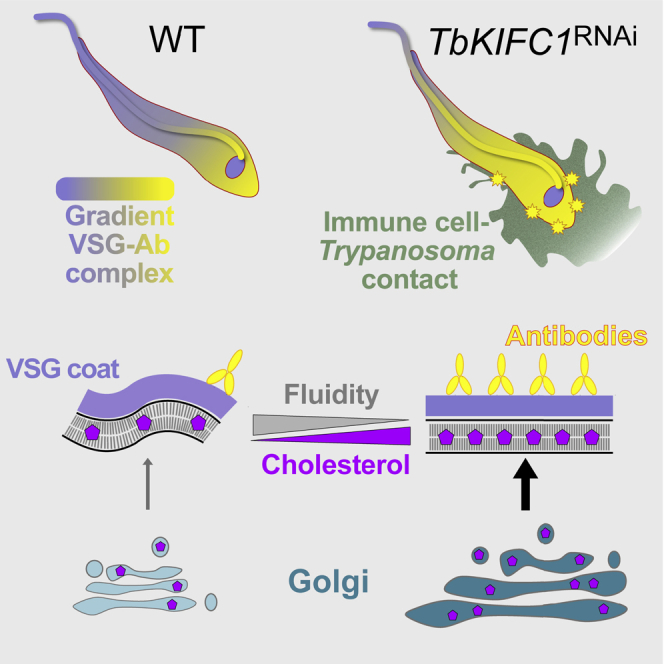

## Introduction

African trypanosomes (prototype: *T. brucei*) are protozoan parasites able to infect a wide variety of mammals, causing important diseases such as Nagana in bovines and sleeping sickness in humans. The life cycle of these parasites requires developmental forms adapted to the *Glossina* fly vector and mammalian host. In the mammalian bloodstream, trypanosomes are confronted with the host immune response. Bloodstream trypanosomes are entirely covered with a dense monolayer of 10^7^ copies of a single type of VSG. Although this dominant antigen triggers an efficient antibody response that clears most parasites, antigenic variation of the VSG ensures that the infection persists long enough for *Glossina* fly-mediated transmission to another host (MacGregor et al., 2012).

In addition to adaptive immunity, primates including humans have developed a specific innate immunity against *T. brucei* (Pays et al., 2014). This immunity results from the trypanolytic activity of the serum protein APOL1, the only secreted member of a family of death-promoting proteins (Hu et al., 2012; Uzureau et al., 2016; Vanhollebeke and Pays, 2006; Wan et al., 2008). The lytic mechanism requires uptake of APOL1, acidic pH-dependent insertion of APOL1 into endosomal membranes, and finally trafficking of these APOL1-containing membranes within the parasite (Pérez-Morga et al., 2005; Vanhamme et al., 2003; Vanwalleghem et al., 2015). APOL1 trafficking to the mitochondrion is a requisite for trypanosome lysis (Vanwalleghem et al., 2015) and involves the kinesin *Tb*KIFC1, which is also associated with the movement of acidocalcisomes (Dutoya et al., 2001; Vanwalleghem et al., 2015). Although *Tb*KIFC1 is more than 1,000-fold more abundant in bloodstream than insect-specific procyclic forms (Dutoya et al., 2001), it is not required for trafficking functions associated with bloodstream forms such as receptor-mediated or fluid phase endocytosis (Vanwalleghem et al., 2015). Moreover, *Tb*KIFC1 down-regulation does not significantly affect parasite growth *in vitro* (Vanwalleghem et al., 2015). Therefore, we investigated the role of this kinesin *in vivo*. This work resulted in the unexpected discovery that *Tb*KIFC1 is instrumental not only in human innate immunity against the parasite but conversely it is also essential for parasite evasion of host adaptive immunity.

## Results

### *Tb*KIFC1 Preferentially Interacts with Cholesterol-Containing Membranes

**Figure 1:**
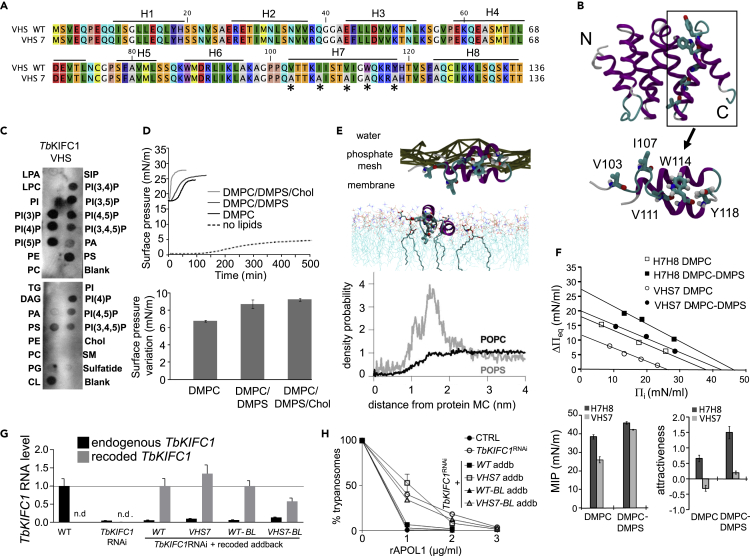
Role of *Tb*KIFC1 in Membrane Trafficking (A) WT and VHS7 mutant sequences of the *Tb*KIFC1 VHS domain, with indication of the different helices (H1–H8). The asterisks point to the mutations in VHS7. (B) Model of the VHS domain, built by homology modeling with 1ELK and 1X5B (PDB codes) as templates. The inset shows the H7-H8 helical hairpin (*Tb*KIFC1 H7H8). Lower panel: model of helices H7H8 highlighting residues V103, I107, V111, W114, and Y118, mutagenized into A in VHS7. (C) Immunodetection of recombinant VHS (0.5 μg/mL) association with various lipids spotted on membrane strips (LPA, lysophosphatidic acid; LPC, lysophosphocholine; PI, phosphatidylinositol; PI(3)P, PI(3)phosphate; PI(4)P, PI(4)phosphate; PI(5)P, PI(5)phosphate; PE, phosphatidylethanolamine; PC, phosphatidylcholine; S1P, sphingosine-1-phosphate; PI(3,4)P2, PI(3,4)bisphosphate; PI(3,5)P2, PI(3,5)bisphosphate; PI(4,5)P2, PI(4,5)bisphosphate; P(3,4,5)P3, PI(3,4,5)trisphosphate; PA, phosphatidic acid; PS, phosphatidylserine; TG, triglyceride; DAG, diacylglycerol; PG, phosphatidylglycerol; CL, cardiolipin; Chol, cholesterol; SM, sphingomyelin; Blank, no lipid). (D) Adsorption of recombinant VHS domain into an air-water interface (without lipids) or into a lipid monolayer with DMPC, DMPC-DMPS (1:1), or DMPC-DMPS-cholesterol (1:1:2). Top panel: kinetics; bottom panel: variation of surface pressure at equilibrium. Data are represented as mean ± SD; n = 3. (E) Molecular dynamics of the interaction between the *Tb*KIFC1 H7H8 peptide and a membrane composed of PC/PS (9:1). In the top section of the figure, the peptide is represented as in (B) (lower panel), and the membrane is represented by a line joining the phosphate groups. The bottom section shows the radial distribution function of POPC (black line) and POPS (red line) around the peptide mass center (MC). (F) Interaction with PS. The adsorption of the H7H8 helices of *Tb*KIFC1 into a lipid monolayer composed of DMPC with or without DMPS (1:1) was evaluated by measuring maximal insertion pressure (MIP, dark gray bar) and attractiveness factor (ΔΠ, light gray bar). The WT and VHS7 mutant version of H7H8 (see A) are compared. MIP corresponds to the x-intercept of the linear regression (inset) of ΔΠ_eq_ (surface pressure at the equilibrium) versus Π_i_ (initial pressure). Data are represented as mean ± SD; n = 3. (G) Relative expression levels of the endogenous or ectopic *TbKIFC1* genes in various *TbKIFC1*^RNAi^ cell lines with or without addback *TbKIFC1*expression (WT or VHS7), as measured by quantitative RT-PCR. Data are represented as mean ± SEM; n = 3. (addb refers to the different recoded *TbKIFC1* addback sequences reinserted in the RNAi line, provided or not with a biotin ligase [BL] tag). (H) Involvement of H7 in APOL1 transport, as measured by trypanolysis as a function of rAPOL1 concentration during incubation with control (CTRL) or *TbKIFC1*^RNAi^ parasites. Data are represented as mean ± SEM; n = 3.

To understand the function of *Tb*KIFC1, we considered first its membrane-interacting potential. The *Tb*KIFC1 N-terminal region contains a vesicular trafficking VPS27/Hrs/STAM (VHS) domain made of eight helices (H1–H8) (Figure 1A). Homology modeling (Arnold et al., 2006) suggested that this VHS is composed of four double-stranded hairpins (Figure 1B). As measured by incubation with membranes containing various spotted lipids, the recombinant VHS domain exhibited binding to anionic lipids, such as phosphatidylinositols (PI) and phosphatidylserine (PS), but no binding to cholesterol alone (Figure 1C). In Langmuir monolayer adsorption experiments (Nasir et al., 2017), this domain adsorbed faster to dimyristoylphosphatidylserine (DMPS) than dimyristoylphosphatidylcholine (DMPC) (initial velocity [IV] = 0.52 ± 0.04 and 0.19 ± 0.00 mN/m/min, respectively), particularly in the presence of cholesterol, which strongly increased VHS binding to the lipids (IV = 1.41 ± 0.13 mN/m/min) (Figure 1D, upper panel). In these experiments, the surface pressure variation at equilibrium, which reflects the penetration power of the VHS domain into a lipid monolayer, was also the highest in the presence of DMPS + cholesterol (Figure 1D, lower panel). Therefore, *Tb*KIFC1 preferentially interacted with membranes containing anionic lipids and cholesterol. Interestingly, helix H7 of the VHS contains a motif predicted to bind cholesterol (CARC: 116 KRYHTV 121) (Fantini and Barrantes, 2013), which may explain the particular affinity of the *Tb*KIFC1 VHS for cholesterol-containing anionic lipids.

Since the C-terminal helical hairpin H7H8 not only contains a possible motif for cholesterol binding but also exhibits a combination of hydrophobic and positively charged amino acids compatible with lipid binding (Figure 1A), the affinity of this hairpin for lipids was assessed by molecular dynamics and docking approaches. Using a coarse grained representation, a 1-μs simulation of a PC/PS system in the presence of H7H8 showed this hairpin to be located at the phospholipid polar head/acyl chain interface, with residues V103, I107, V111, W114, and Y118 of helix H7 facing the membrane interior (Figure 1B, lower panel; Figure 1E, upper panel). Helix H7 interacted better with PS than PC (Figure 1E, lower panel). Using docking simulation, the relative interaction energy of H7H8 was found to be −80 and −50 kcal/mol for assembly with five PS and five PC molecules, respectively. Langmuir monolayer experiments confirmed that PS favors the insertion of H7H8 into model membranes, since both maximal insertion pressure (MIP) and attractiveness factor (ΔΠ) of the synthetic H7H8 peptide were increased with PS (Figure 1F). In contrast, a mutant H7H8 peptide termed VHS7, where the hydrophobic residues predicted to contact the membrane were replaced by alanine (V103A/I107A/V111A/W114A/Y118A), had significantly lower MIP and ΔΠ values than the wild-type (WT) version (Figure 1F). Accordingly, the mutant VHS7 domain exhibited much slower adsorption to lipids than the WT, and the preferential binding to PS and cholesterol relative to PC was strongly reduced (IV = 0.07 ± 0.00, 0.10 ± 0.03 and 0.09 ± 0.01 mN/m/min for DMPC, DMPC/DMPS, and DMPC/DMPS/cholesterol, respectively) (Figure S1). We conclude that helix H7 allows *Tb*KIFC1 VHS binding to PS- and cholesterol-containing membranes.

### *Tb*KIFC1 Transports Endosomal Membranes

The role of *Tb*KIFC1 in membrane trafficking was investigated in trypanosomes expressing the wild-type (WT) kinesin or its mutant VHS7 version. In these experiments, APOL1-mediated killing of *TbKIFC1*^RNAi^ cells was measured following addback of recoded (thus, RNAi-insensitive) versions of the *Tb*KIFC1 gene (Vanwalleghem et al., 2015), either as WT or VHS7 (see Figures 1G and S2, for evidence of *Tb*KIFC1 down-regulation and addback re-expression in the RNAi line). As expected (Vanwalleghem et al., 2015), addback of the recoded WT version restored normal trypanolytic activity following knockdown of the endogenous *Tb*KIFC1 transcript (Figure 1H). However, addback of the recoded VHS7 mutant did not (Figure 1H). This result indicated that the VHS7 mutant, which interacts poorly with membranes, has lost its APOL1 trafficking activity.

**Figure 2:**
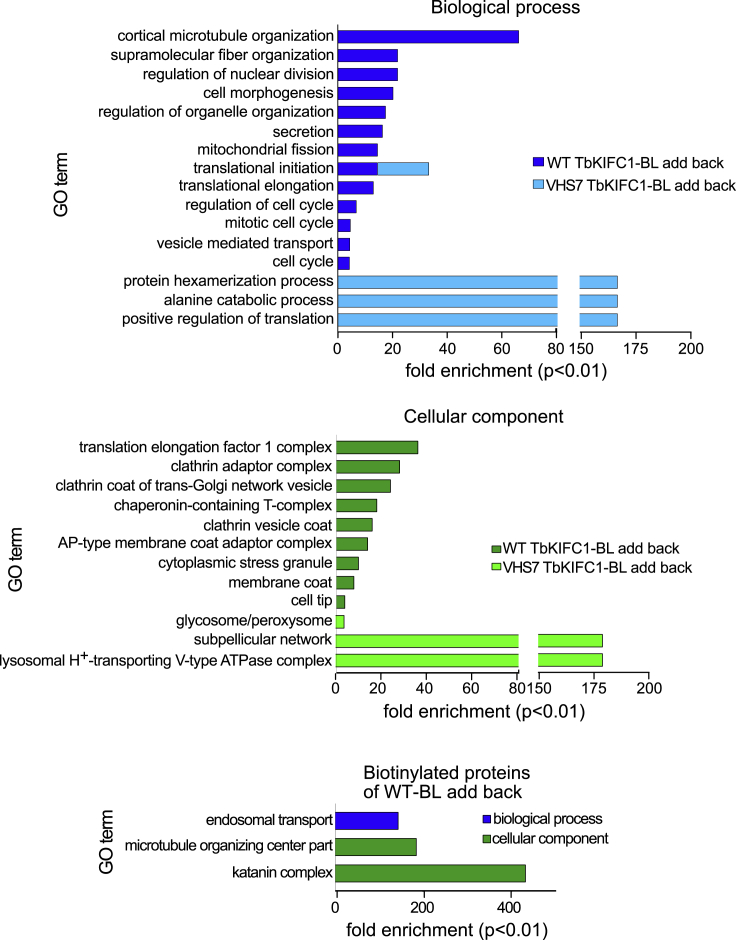
Proteins Associated with *Tb*KIFC1 The proteome associated with BL-conjugated *Tb*KIFC1, either WT or VHS7 mutant, was characterized by mass spectrometry of proteins bound to streptavidin agarose beads. These proteins were analyzed for their relationship with cellular components and biological activity in TriTryp database through Gene Ontology terms enrichment. See Table S1 for the list of identified proteins.

Biotin ligase (BL)-conjugated versions of addback *Tb*KIFC1, both WT and VHS7, were employed to identify proteins proximal to the kinesin, following chromatography of cellular extracts on streptavidin (Roux et al., 2012) (see Figures 1G and S2, for evidence of proper expression of BL-conjugated *Tb*KIFC1). As expected, this screen identified several tubulin-related components, in particular two proteins biotinylated by the *Tb*KIFC1-BL conjugate, a tubulin polymerization promoting factor (Tb927.4.2740) and a tubulin-associated AIR9-like protein (Tb927.11.17000) (Table S1). In addition, the WT *Tb*KIFC1-BL-associated proteome, but not the VHS7 version, included several proteins involved in vesicular membrane traffic, in particular the vacuolar protein sorting-associated protein 4 (VPS4) (Tb927.3.3280) and the katanin p60-like protein 2 (Tb927.10.1210), which contains a VPS4 C-terminal oligomerization domain and is predicted to transport endosomes (https://tritrypdb.org/tritrypdb/app/record/gene/Tb927.10.1210). The p60-like katanin probably contacted *Tb*KIFC1, since this protein was biotinylated by the *Tb*KIFC1-BL conjugate (Table S1). Thus, proteins involved in endosomal traffic were significantly enriched in the fraction isolated from cells expressing BL-conjugated WT *Tb*KIFC1 but not VHS7-BL (Figure 2 and Table S1). We conclude that the H7 helix of *Tb*KIFC1 VHS is involved in both APOL1 and endosomal membrane trafficking.

### *Tb*KIFC1 Is Required for Evasion of the Parasite from Adaptive Immunity

**Figure 3:**
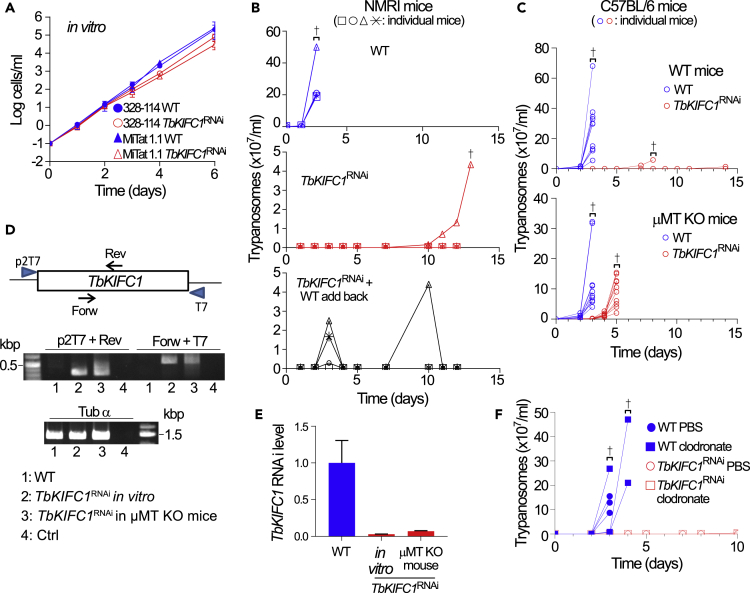
Role of *Tb*KIFC1 in Parasite Growth (A) Cumulative *in vitro* growth of two independent *T. brucei* lines, either WT or *TbKIFC1*^RNAi^. Data are represented as mean ± SD; n = 3. (B) Trypanosome parasitemia in four NMRI mice infected intraperitoneally with 10^5^*T. brucei* 328-114 bloodstream forms (WT), *TbKIFC1*^RNAi^, or *WT* addback *TbKIFC1*^RNAi^ parasites. The infection was monitored for each individual mouse (n = 5). (C) Parasitemia in 10 WT or B cell-defective (μM KO) C57BL/6 mice infected intraperitoneally with WT or *TbKIFC1*^RNAi^ parasites. The infection was monitored for each individual mouse (n = 2). (D) PCR detection of the *TbKIFC1* RNAi construct in the genomic DNA of WT *T. brucei*, or in *TbKIFC1*^RNAi^ trypanosomes grown either *in vitro* or in μM KO mice (day 5) (CTRL = no DNA). Data are represented as mean ± SD; n = 3. (E) RT-PCR detection of *TbKIFC1* RNA in WT *T. brucei*, or in *TbKIFC1*^RNAi^ trypanosomes grown either *in vitro* or in μM KO mice (day 5). Data are represented as mean ± SD; n = 3. (F) Parasitemia in three C57BL/6 mice infected intraperitoneally with WT or *TbKIFC1*^RNAi^ parasites after prior injection of liposomes containing either phosphate buffer (PBS) or clodronate. The infection was monitored for each individual mouse (n = 2).

Since expression of *Tb*KIFC1 is restricted to bloodstream forms, we considered the possible involvement of this kinesin in functions linked to the VSG and immune evasion. These studies involved generating cloned *TbKIFC1*^RNAi^ cell lines in MiTat 1.1 parasites because of the availability of anti-MiTat 1.1 VSG IgGs, IgMs, and immune serum (O'Beirne et al., 1998). Both the previous (Vanwalleghem et al., 2015) and these new *TbKIFC1*^RNAi^ cell lines exhibited only slightly reduced *in vitro* growth rate relative to WT parasites (Figure 3A). In striking contrast, the *TbKIFC1*^RNAi^ trypanosomes were unable to infect mice, whereas WT parasites killed the animals within 3 days (Figures 3B and 3C). The detection of parasites in one mouse after day 10 was probably due to loss of knockdown of *Tb*KIFC1 in the parasites. Addback of recoded WT *Tb*KIFC1 (Vanwalleghem et al., 2015) into the *TbKIFC1*^RNAi^ trypanosomes partially restored parasite infectivity, as three of four mice infected with these cells developed a parasitemia (Figure 3B). The partial nature of this restoration of infectivity probably reflected the low expression of recoded *Tb*KIFC1 in these cells relative to true WT levels (see Figure 6E in Vanwalleghem et al., 2015). The growth of *TbKIFC1*^RNAi^ cells *in vitro* but not *in vivo* indicated a functional requirement for *Tb*KIFC1 for growth in the mammalian host. Significantly, inoculation of *TbKIFC1*^RNAi^ trypanosomes in B cell-deficient mice (μMT KO) allowed full recovery of infectivity after a delay of 3 days (Figure 3C), despite the fact that parasites growing in these mice still exhibited strong down-regulation of *Tb*KIFC1 linked to the conservation of the RNAi construct in these cells (Figures 3D and 3E). Thus, the infectivity of *TbKIFC1*^RNAi^ cells was compromised because of the host antibody response, and *Tb*KIFC1 was required to overcome this response. Interestingly, suppression of phagocytic activity was not sufficient to rescue the virulence defect in *TbKIFC1*^RNAi^ cells, as these cells were unable to infect mice treated with clodronate liposomes to deplete mononuclear phagocytes (Figure 3F).

### *Tb*KIFC1 Is Required for Clearance of VSG/Antibody Complexes

**Figure 4:**
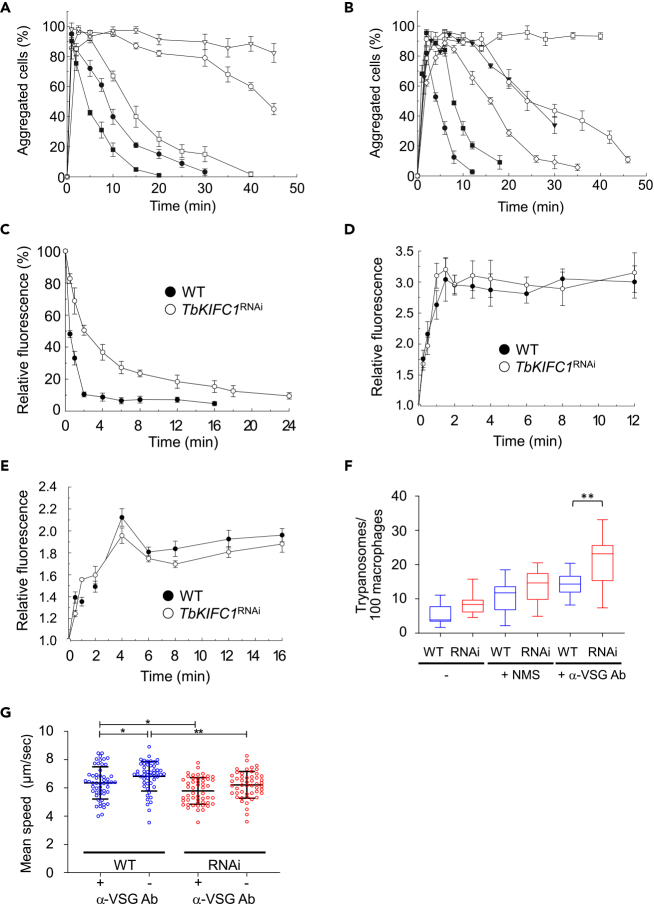
Role of *Tb*KIFC1 in Surface Trafficking of VSG/Antibody Complexes (A) Disaggregation of WT or clone 1 *TbKIFC1*^RNAi^ cells following addition of anti-VSG IgGs as follows: WT: ■, 1 μg/mL; ●, 2 μg/mL; *TbKIFC1*^RNAi^ cells: □, 1 μg/mL; ◯, 2 μg/mL; ▽, 3 μg/mL. Data are represented as mean ± SD; n = 3. (B) Disaggregation of WT and *TbKIFC1*^RNAi^ cells following addition of purified anti-VSG IgMs as follows: WT: ▼, 40 μg/mL; ■, 16 μg/mL; ●, 8 μg/mL. *TbKIFC1*^RNAi^ cells: □, 16 μg/mL; ◯, 8 μg/m; ◇, 4 μg/mL. Data are represented as mean ± SD; n = 3. (C) Clearance of surface-bound antibodies as determined by flow cytometry of WT and clone 1 *TbKIFC1*^RNAi^ cells incubated with anti-VSG immune serum. The data are expressed as percent median fluorescence intensity detected relative to time zero. Data are represented as mean ± SD; n = 3. (D) Internalization of surface-biotinylated VSG. After incubation at 37°C for various times, internalization of surface-biotinylated WT in clone 1 *TbKIFC1*^RNAi^ cells was determined by flow cytometry. The data are expressed as percent median fluorescence intensity detected relative to time zero. Data are represented as mean ± SD; n = 3. (E) Return of internal biotinylated VSG to the surface. After incubation at 37°C for 15 min, membrane traffic of surface-biotinylated WT and *TbKIFC1*^RNAi^ cells (clone 1) was cold-stopped and the remaining surface biotin was removed with reduced glutathione. The appearance of surface biotin was determined by flow cytometry after incubation for various times at 37°C. The data are expressed as in (D). Data are represented as mean ± SD; n = 3. (F) Binding and uptake of WT and *TbKIFC1*^RNAi^ parasites by murine RAW264.7 macrophages, after parasite incubation or not for 30 min with either 10% normal mouse serum (NMS) or anti-MITat 1.1 VSG antibodies. Data are represented as mean ± SD; n = 3. One-way ANOVA, Sidak's multiple comparison test ∗∗p < 0.01. (G) Mobility of WT and *TbKIFC1*^RNAi^ trypanosomes after incubation or not with anti-MITat 1.1 VSG antibodies. The data are from a representative experiment. Data are represented as mean ± SD; n = 3. ANOVA/Dunn's multiple comparison test ∗p < 0.01; ∗∗p < 0.05

VSG-antibody complexes are rapidly cleared from the surface of trypanosomes, and it has long been speculated that this clearance might have relevance for parasite survival in the mammalian host (Cheung et al., 2016; Engstler et al., 2007; O'Beirne et al., 1998; Barry, 1979). This clearance has been characterized by monitoring parasite disaggregation after incubation with purified anti-VSG antibodies (O'Beirne et al., 1998). Significantly, disaggregation of trypanosomes treated with anti-VSG IgGs was far slower in *TbKIFC1*^RNAi^ cells (in three independent lines) than in WT cells, and the magnitude of the effect increased with the antibody level (Figures 4A, S3A, and S3B). Since IgM is the first antibody to appear after initial exposure to VSG (O'Beirne et al., 1998) and IgM is cleared more rapidly than IgG (Engstler et al., 2007), the effect of knockdown of *Tb*KIFC1 on clearance of anti-VSG IgM was examined (Figure 4B). In all cases the disaggregation of WT trypanosomes was faster than that of *TbKIFC1*^RNAi^ cells. Moreover, the effect for IgMs appeared to be more pronounced than for IgGs. For example, WT parasites and *TbKIFC1*^RNAi^ parasites treated with IgMs (8 μg/mL) had a T_1/2_ for disaggregation of 5 and 25 min, respectively. At higher levels of IgMs (16 μg/mL), *TbKIFC1*^RNAi^ cells failed to disaggregate during the course of the assay, whereas WT cells disaggregated with a T_1/2_ of 10 min (Figure 4B). However, at lower levels of IgMs (4 μg/mL), clearance of antibody was apparently so fast in WT parasites that no aggregation could be observed (not shown), but aggregation of *TbKIFC1*^RNAi^ cells was obvious (Figure 4B). The same results were obtained with two other *TbKIFC1*^RNAi^ clones (Figure S3C).

A more direct assay of antibody clearance from the surface of individual cells, by flow cytometry, confirmed a substantial reduction in clearance in *TbKIFC1*^RNAi^ cells (Figures 4C, S4A, and S4B). Immunofluorescence microscopy provided visual confirmation of this delay (Figure S4C).

In *VSG*^RNAi^ parasites (Cheung et al., 2016), clearance of anti-VSG antibodies was also compromised owing to the decreased packing density of VSG on the surface. However, there was no obvious difference in the level of VSG expressed in *TbKIFC1*^RNAi^ lines relative to WT cells (Figure S5A). Moreover, the pattern and relative intensity of the labeling of VSG and other proteins after surface biotinylation was essentially identical in *TbKIFC1*^RNAi^ and WT cells (Figure S5B). Finally, there was no significant difference in the internalization of biotinylated VSG in *TbKIFC1*^*RNAi*^ compared with WT cells (Figure 4D), nor was there a significant difference in the return of internalized biotinylated proteins back to the surface (Figure 4E).

Therefore, knockdown of *Tb*KIFC1 only affected the surface mobility of antibody-VSG complexes but not the expression, surface location, or trafficking of the VSG itself.

### *Tb*KIFC1 Influences Both Parasite Capture by Macrophages and Swimming Speed

To evaluate the influence of antibody persistence on the surface in *TbKIFC1*^RNAi^ parasites, trypanosomes were co-incubated with macrophages after opsonization with normal mouse serum (NMS) or anti-VSG antibodies. *TbKIFC1*^RNAi^ parasites exhibited higher antibody-mediated capture by macrophages than WT trypanosomes (Figures 4F and S6A). Knockdown of *Tb*KIFC1 also resulted in a slight reduction in the trypanosome swimming speed (Figure 4G). This effect was not linked to detectable structural changes in the flagellum, such as in the flagellar axoneme and associated structures, paraflagellar rod (PFR), and flagellum associated zone (FAZ) (Figure S6B). There is evidence that motility is essential for infectivity (Shimogawa et al., 2018), so even though the defect in *TbKIFC1*^RNAi^ parasites is very slight, it may explain their increased capture by macrophages even in the absence of specific antibodies (Figure 4F), as well as the slower development of the infection in μMT KO mice (Figure 3C).

### *Tb*KIFC1 Influences Plasma Membrane Fluidity

**Figure 5:**
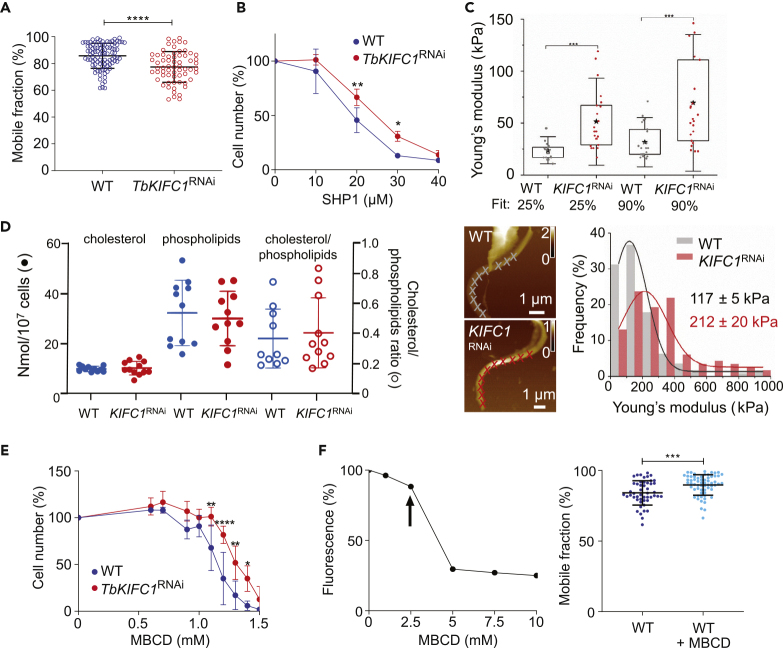
Role of TbKIFC1 in Plasma Membrane Dynamics (A) VSG mobility in the plasma membrane of WT and *TbKIFC1*^RNAi^ trypanosomes, as measured by FRAP. Data are represented as mean ± SD; n = 3. Mann-Whitney test ∗∗∗∗p < 0.0001. (B) Sensitivity of WT and *TbKIFC1*^RNAi^ trypanosomes to the toxicity of the membrane-stiffening peptide SHP1. Data are represented as mean ± SD; n = 3. Two-way ANOVA, Sidak's multiple comparison test ∗p < 0.05; ∗∗p < 0.01. (C) Atomic force microscopy (AFM) measurements of trypanosome surface mechanical properties. Upper panel: Young's modulus values of the cellular body membrane of individual WT (gray dots) and *TbKIFC1*^RNAi^ (red dots) trypanosomes assessed from AFM multiparametric imaging. Values were extracted from force curves using Hertz fits up to 25% (mainly plasma membrane contribution) and 90% (mainly microtubules contribution) of the maximum applied force. Bottom left: AFM height images of the flagellum of WT and *TbKIFC1*^RNAi^ trypanosomes. Crosses indicate the location along the flagellum where single force-distance curves were extracted and fitted with the Hertz model to obtain the Young's modulus (scales in μm). Bottom right: histograms and corresponding Gaussian fit revealing the elastic Young's modulus of WT and *TbKIFC1*^RNAi^ flagella. Data are representative of n ≥ 30 cells per condition. Data are represented as mean ± SD; n = 6. One-way ANOVA test ∗∗∗p < 0.001. (D) Comparison of cholesterol and phospholipid amounts in WT and *TbKIFC1*^RNAi^ trypanosomes. Data are represented as mean ± SD; n = 3. (E) Trypanosome resistance to 18-h incubation with increasing amounts of MBCD. Data are represented as mean ± SD; n = 3. Two-way ANOVA, Sidak's multiple comparisons test ∗p < 0.05; ∗∗p < 0.01; ∗∗∗∗p < 0.0001. (F) Effect of MBCD on cholesterol levels and plasma membrane fluidity of WT parasites. Left panel: the MBCD concentration necessary to obtain 10% reduction of cholesterol, as measured by flow cytometry of parasites incubated with Top Fluor cholesterol, was determined to be 2.5 mM (arrow). Right panel: FRAP analysis of WT parasites pre-incubated or not for 30 min with 2.5 mM MBCD. Data are represented as mean ± SD; n = 3. T test ∗∗∗p < 0.05.

The effects of *TbKIFC1*^RNAi^ on trypanosome speed and surface mobility of antibody-VSG complexes were consistent with a change in the physical properties of the surface membrane, for example, membrane fluidity. In order to evaluate the relative fluidity of the membrane, the kinetics of membrane fluorescence recovery after photobleaching (FRAP) were measured on *TbKIFC1*^RNAi^ and WT cells. This recovery was slower in *TbKIFC1*^RNAi^ trypanosomes, suggesting that the fluidity of the plasma membrane, and hence VSG mobility, is lower in these cells (Figure 5A). Accordingly, *TbKIFC1*^RNAi^ trypanosomes were more resistant to the SHP-1 peptide, a membrane stiffening peptide that inserts preferentially into fluid membranes (Harrington et al., 2010) (Figure 5B). Finally, multiparametric atomic force microscopy (Krieg et al., 2019) showed an increase in the rigidity of the *TbKIFC1*^RNAi^ surface, on both the cell body (Figure 5C, upper panel) and the flagellum (Figure 5C, lower panel). In both WT and *TbKIFC1*^RNAi^ cells the flagellar surface was more rigid than the cellular body, most likely due to the higher cholesterol content of the flagellar membrane (Sharma et al., 2017). Taken together, these observations indicated that knockdown of *Tb*KIFC1 results in a less fluid and more rigid plasma membrane.

### *Tb*KIFC1 Reduces the Cholesterol Level of the Plasma Membrane

Cholesterol content is a key modulator of eukaryotic plasma membrane fluidity, with higher levels contributing to a less fluid and more rigid membrane. *T. brucei* bloodstream forms are auxotrophic for cholesterol, which is acquired from the mammalian host by receptor-mediated endocytosis (Smith and Bütikofer, 2010). Endocytic activity is not affected by *Tb*KIFC1 down-regulation (Vanwalleghem et al., 2015), and not surprisingly, neither the total cellular cholesterol content nor cholesterol/phospholipid ratio were affected by *Tb*KIFC1 depletion (Figure 5D). However, the cholesterol content of the plasma membrane of *TbKIFC1*^RNAi^ trypanosomes appeared to be higher than that of WT cells. For example, *TbKIFC1*^RNAi^ trypanosomes were more resistant to the toxic effect of the cholesterol-removing agent methyl-β-cyclodextrin (MBCD) (Figure 5E), which can increase membrane fluidity following even a modest reduction of cholesterol, as shown by FRAP experiments (Figure 5F).

**Figure 6:**
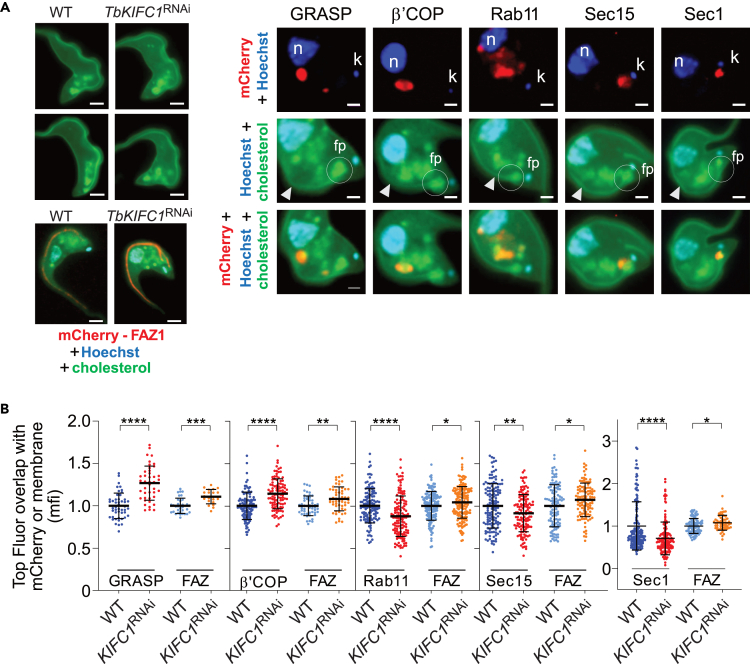
Role of TbKIFC1 in Cholesterol Trafficking to the Cell Surface (A) Left panel: global fluorescence of WT and *TbKIFC1*^RNAi^ parasites after incubation for 60 min in Top Fluor Cholesterol, and relative co-localization of the flagellum-associated Top Fluor fluorescence with mCherry-tagged FAZ (scale bar = 2 μm). Right panel: intracellular localization in WT trypanosomes of Top Fluor cholesterol and different markers of intracellular traffic to the plasma membrane, tagged with mCherry (GRASP and β′COP for the Golgi, Rab11 for recycling endosomes and exocyst, Sec15 and Sec1 for the exocyst). The arrows designate the flagellum-associated Top Fluor labeling, apparently co-localizing with the FAZ (scale bar = 1 μm; n, nucleus and k, kinetoplast, both stained in blue with Hoechst; fp, flagellar pocket). (B) Relative levels of cholesterol mean fluorescence intensity (mfi) after 60 min Top Fluor cholesterol uptake without prior cholesterol starvation, measured as either co-localizing with the FAZ as marker of the cell surface or co-localizing with fluorescence of mCherry conjugated with different intracellular markers (Golgi GRASP and β′COP, recycling endosomes/exocyst Rab11 or exocyst Sec15 and Sec1). Top Fluor fluorescence was measured within the exact surface of mCherry fluorescence. As internal reference for each trypanosome preparation, Top Fluor measurement in the FAZ region (similar surface in the different cells) was performed together with each of the mCherry measurements. Data are represented as mean ± SD; n = 3. Mann-Whitney test ∗p < 0.05; ∗∗p < 0.01; ∗∗∗p < 0.001; ∗∗∗∗p < 0.0001.

**Figure 7:**
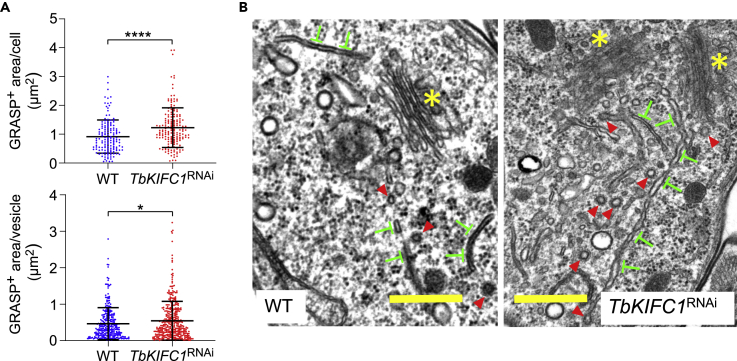
Cholesterol Traffic and Golgi Structure in *TbKIFC1*^RNAi^ Trypanosomes (A) Size of GRASP^+^ compartments (by vesicle or by cell) in WT and *TbKIFC1*^RNAi^ trypanosomes. Data are represented as mean ± SD; n = 3. Mann-Whitney test ∗p < 0.05; ∗∗∗∗p < 0.0001. (B) Transmission electron microscopy analysis of WT and *TbKIFC1*^RNAi^ trypanosomes. Yellow asterisks and red arrows, respectively, label the *cis*-Golgi and clathrin-coated vesicles, and inverted green “T"s designate TGN-issued secretory vesicles that appear unprocessed in *TbKIFC1*^RNAi^ trypanosomes, therefore accumulating and increasing in size in these cells (scale bar = 500 nm).

The relative distribution of fluorescent cholesterol (Top Fluor) was employed to assess the levels of cholesterol co-localizing with trafficking compartments involved in vesicular routing to the cellular surface, between the nucleus-proximal Golgi and the kinetoplast-proximal flagellar pocket (Figure 6A). To measure cholesterol associated with the plasma membrane, we quantified the fluorescence of the membrane portion co-localizing with the mCherry-FAZ1 fusion protein (Figure 6A). This fluorescence was around 10% higher in *TbKIFC1*^RNAi^ cells than in WT cells, and this measurement was used as a control in all subsequent experiments. Quantitative analysis of cholesterol fluorescence in vesicular compartments revealed an altered distribution of cholesterol in *TbKIFC1*^RNAi^ cells relative to WT trypanosomes (Figure 6B). Cholesterol in *TbKIFC1*^RNAi^ cells appeared to be more prominently associated with the Golgi (around 20%–25% increase), using as Golgi markers the General Receptor for phosphoinositides 1-Associated Scaffold Protein (GRASP) and the coatomer β’ (β′ COP: see Figure S7 for β′ COP association with the Golgi). In contrast, it was less prominent (around 10% decrease) in the post-Golgi compartment involved in exocytosis (Rab11, Sec15 and Sec1 markers: Gabernet-Castello et al., 2011) (Figure 6). Thus, knockdown of *Tb*KIFC1 leads to cholesterol accumulation in both Golgi and plasma membrane (as measured here along the FAZ), together with reduction of cholesterol associated with exocytosis. These data suggest that *Tb*KIFC1 plays a role in the traffic of cholesterol-containing membranes within the parasite and that loss of *Tb*KIFC1 causes an increase in the cholesterol content of the Golgi and surface membranes. Since cholesterol is known to inhibit membrane fission and promote membrane fusion (Najafinobar et al., 2016; Yang et al., 2016), the accumulation of cholesterol in Golgi membranes of *TbKIFC1*^RNAi^ cells might account for the observed enlargement of Golgi cisternae in these cells (Figure 7A). Given the inhibition of fission in the secretory pathway, the TGN compartment increased in size, giving rise to unresolved flat cisternae and accumulation of clathrin-coated pits (Figure 7B). In the plasma membrane, the increase of cholesterol content is expected to reduce membrane fluidity, with major implications for growth in the mammalian host, but not *in vitro*.

## Discussion

The kinesin *Tb*KIFC1 is a surprising protein for many reasons. Originally linked with acidocalcisome traffic (Dutoya et al., 2001), it was later shown to play a key role in human innate immunity to *T. brucei* because of its function in the trafficking of the host-derived trypanolytic factor APOL1 within the parasite (Pays et al., 2014; Vanhamme et al., 2003; Vanhollebeke et al., 2008; Vanwalleghem et al., 2015). These functions fit with the widespread distribution of *Tb*KIFC1 throughout the cell, along with some concentration in the posterior region associated with vesicle traffic (Figure S2). Here we report another, unexpected role for *Tb*KIFC1: it is essential for parasite evasion of the mammalian adaptive immune response. Specifically, we propose that *Tb*KIFC1-mediated intracellular traffic is required to maintain appropriate levels of cholesterol in the surface membrane and a corresponding level of membrane fluidity that allows for a rapid clearance of VSG-antibody complexes from this surface. The VSG surface coat of African trypanosomes operates close to its molecular crowding threshold, yet the lateral mobility and mobile fraction of VSG in this membrane are very high (Hartel et al., 2016). Both these parameters depend on the composition and physical properties of the surface membrane (Hartel et al., 2016), and changes in the cholesterol content of this membrane could dramatically affect the mobility of VSG-antibody complexes. A stiffer, less fluid surface membrane in *Tb*KIFC1 knockdown parasites results in a slower clearance of these complexes from the trypanosome surface and consequently they cannot infect the mammalian host unless antibody synthesis is compromised. Our data demonstrate that clearance of surface-bound antibodies by trypanosomes is physiologically relevant, being required for mammalian infectivity, and that high VSG mobility in the surface membrane is critical for rapid clearance. Evidence suggests that trypanosome motility may drive directional movement of VSG-antibody complexes on the surface (Cheung et al., 2016; Engstler et al., 2007; O'Beirne et al., 1998), so the motility defect present in *TbKIFC1*^RNAi^ cells may contribute to slower clearance of VSG-antibody complexes. The idea of hydrodynamic flow-mediated sorting of antibody-VSG complexes (Engstler et al., 2007) is attractive, but an essentially immotile trypanosome, the dynein LC1-double knock-in (DKI) mutant, can clear VSG-IgG complexes from the surface at the same rate as WT parasites (Shimogawa et al., 2018). In the case of *TbKIFC1*^RNAi^ cells the motility effect appears very minor, so the slower rate of clearance of VSG-antibody complexes is probably due mainly to the decreased VSG mobility in a surface membrane with a higher cholesterol content. Interestingly, as observed for *TbKIFC1*^RNAi^ cells, the immotile mutants of Shimogawa et al. (2018) could not infect mice unless antibody synthesis was compromised. Therefore, *TbKIFC1*^RNAi^ cells are trypanosomes with essentially normal motility but impaired clearance of VSG-antibody complexes, whereas LC1-DKI cells are the reverse, immotile trypanosomes that clear surface-bound antibodies normally. It seems that clearance of surface-bound antibodies and motility are separate virulence factors in trypanosomes and both are required to overcome the host adaptive immune response.

The first responder here is IgM, and clearance of this immunoglobulin from the surface seems particularly important for trypanosomes. Anti-VSG IgM antibodies are detectable, albeit at low levels, as early as 3 days after infection (O'Beirne et al., 1998). Therefore, removal of VSG-IgM complexes must be important early in the infection. This may explain why no parasites were detected in *TbKIFC1*^RNAi^ infections of WT mice at day 3. Moreover, it is striking that IgMs are cleared from the surface of trypanosomes far faster than IgGs (Engstler et al., 2007) and the deleterious effect of *Tb*KIFC1 knockdown on clearance of antibodies appears more pronounced for IgMs than IgGs. It is tempting to speculate that clearance of immunoglobulins from the surface of trypanosomes is primarily designed to ensure that IgMs spend as little time on the surface as possible. One reason might be that IgMs are more efficient than IgGs at activating the complement system and failure to clear IgMs efficiently renders the trypanosome vulnerable to complement attack. There is indirect evidence to support this view, as the virulence defect in *TbKIFC1*^RNAi^ cells was not rescued in mice treated with clodronate liposomes to deplete mononuclear phagocytes. Interestingly too, non-dividing stumpy bloodstream forms, which clear VSG-antibody complexes even faster than slender forms because of their intrinsically higher rate of endocytosis (Engstler et al., 2007), are much less sensitive to complement-mediated lysis than slender forms (McLintock et al., 1993). So, rapid clearance of surface antibodies may have evolved to protect the trypanosome from IgM-mediated complement lysis.

Why does VSG switching not obviate the need for clearance of surface-bound antibodies in order to infect a mammalian host? The most likely reason is that VSG is an extremely abundant and stable protein, 10^7^ copies per cell with a half-life of over 200 h (Engstler et al., 2004; Seyfang et al., 1990). Thus, previously expressed VSG could persist for some time on the surface of trypanosomes that have switched VSG. This issue was investigated by Pinger et al. (2018), using trypanosome clones expressing two VSGs at varied ratios, representing parasites at multiple stages of VSG coat replacement. These parasites were used for *in vivo* infection assays in mice previously immunized against the titrating VSG. This study indicated that the previously expressed VSG could persist for some time on the surface of trypanosomes after a switch, but that there was only a narrow time period post switch when the switchers remained vulnerable to IgMs. This period was estimated to be up to 29 h after the switch event, when the level of the previously expressed VSG remained above somewhere between 1.3% and 7.6% of the total surface VSG. They proposed that below this antigen density the efficacy of IgM binding is reduced below a threshold level. Interestingly, the experiments of Pinger et al. (2018) were performed in mice immunized against the titrating VSG and so, the assay examined the effect of a high titer of IgM antibody as VSG antigen density decreased. A more natural situation would be increasing antibody levels as the level of the previously expressed VSG in the switcher decreases, and it is here that clearance of surface-bound IgM is likely to have a key role. Clearance assays show that, as antibody titers rise, clearance of surface-bound antibody gets progressively slower until eventually the system is overwhelmed because of the amount of IgM bound to VSG, which is what happens with the non-switchers. However, in a natural infection, as the level of IgMs increases over time the level of the previously expressed VSG in switchers is decreasing, so IgM-VSG complexes can still be efficiently cleared from these trypanosomes. Therefore, antibody clearance, and specifically clearance of surface-bound IgMs, protects trypanosomes at low antibody levels and high surface antigen density, e.g., early in the infection or shortly after a VSG switch, and also as the antibody levels increase and antigen density decreases as switchers divide, e.g., during the period from about 29 h after the switch until complete replacement of the VSG coat at 5–6 days. We propose that, when clearance of antibody-VSG complexes is compromised, as occurs in *TbKIFC1*^RNAi^ cells, both these groups become vulnerable to immune attack and therefore infection cannot develop.

It is important to note that the movement of VSG-antibody complexes is slower in *TbKIFC1*^RNAi^ cells because, as shown by photobleaching experiments, the lateral mobility of all VSGs is lower in these cells. Unlike non-bound VSG, VSG-antibody complexes are selectively sorted and concentrated toward the flagellar pocket/posterior end of the cells, in a process that is slowed down in *TbKIFC1*^RNAi^ cells because the membrane is less fluid. Internalization of VSG is not dependent on the mobility of an individual VSG but rather depends on the rate of membrane uptake from the surface through the flagellar pocket. As the VSG density appears to be unchanged in *TbKIFC1*^RNAi^ cells, the bulk VSG uptake is not expected to be affected in these cells, as indeed observed.

Although the known role of *Tb*KIFC1 was limited to the transport of acidocalcisomes and APOL1 (Dutoya et al., 2001; Pays et al., 2014; Vanwalleghem et al., 2015), the widespread intracellular distribution of this kinesin (Dutoya et al., 2001; Figure S2) suggested an important function in intracellular traffic. So how might this intracellular activity of *Tb*KIFC1 affect surface membrane fluidity/rigidity? Our data indicated that knockdown of *Tb*KIFC1 leads to higher cholesterol levels in both Golgi and plasma membrane, with a concomitant reduction in cholesterol levels in membranes associated with exocyst components such as Sec15 and Sec1. Therefore, we suggest that *Tb*KIFC1 is involved in the homeostasis of membrane cholesterol content and that the absence of this kinesin alters the balance of cholesterol flow between the intracellular and surface membranes.

Several observations are consistent with a role for *Tb*KIFC1 in intracellular cholesterol traffic. First, the *Tb*KIFC1 VHS domain interacted much better with anionic membranes when cholesterol was present. In particular, helix 7 of the VHS, which appears to interact directly with membranes, contains a motif for cholesterol binding (CARC) (Fantini and Barrantes, 2013). This motif is known to exhibit high affinity for cholesterol when the latter is present in the outer leaflet of membrane bilayers (Di Scala et al., 2017), which corresponds to the topology of the VHS/membrane interface. Moreover, the essential tyrosine of this CARC motif belongs to the residues whose mutations in the VSH7 mutant resulted in the loss of *Tb*KIFC1 membrane-trafficking activity. Second, the VHS domain of *Tb*KIFC1 may influence endosome trafficking, as suggested by observations made on the VHS domain of the adaptor protein Target of Myb protein 1 (TOM1), whose structure was used here as template for the modeling of the *Tb*KIFC1 VHS given its similarity with this domain. Indeed, the TOM1 VHS, which binds to phosphoinositides like the *Tb*KIFC1 VHS, was reported to influence endosome trafficking (Boal et al., 2015). Third, *Tb*KIFC1 is clearly involved in the intracellular transport of APOL1 (Vanwalleghem et al., 2015), which is associated with cholesterol in the high-density lipoproteins (HDL3) particles taken up into the parasite by receptor-mediated endocytosis (Vanhollebeke et al., 2008). It is possible that this cholesterol remains associated with APOL1 in endosomal membranes and is subsequently trafficked by *Tb*KIFC1 within the parasite. Finally, within the *Tb*KIFC1-proximal proteome we identified *Tb*VPS4 together with a katanin containing a VPS4 oligomerization domain, and VPS4 is known to be involved in endosomal cholesterol trafficking in both mammals and protozoa (Bishop and Woodman, 2000; Du et al., 2013; Yang et al., 2004). Therefore, we propose that *Tb*KIFC1 is involved in moving membranes rich in cholesterol, most likely from endosomal compartments to other intracellular membranes. Suppression of this traffic would cause an increased flow of cholesterol to the surface through other routes, such as the recycling pathway from the Golgi. This redistribution is expected to give rise to a less fluid, stiffer surface membrane, as we observed in *TbKIFC1*^RNAi^ cells.

It has long been recognized that preventing or disruption of antigenic variation represents an ideal way to prevent/treat trypanosomiasis as the parasites would then become vulnerable to the host immune response. Our data suggest that drugs designed to inhibit *Tb*KIFC1 activity and disrupt clearance of surface bound antibodies could be a simpler way to achieve the same goal.

### Limitations of the Study

This work did not allow fully detailed description of the pathway of cholesterol trafficking in the trypanosome, as we only document that *Tb*KIFC1 knockdown alters the balance of cholesterol flow between the intracellular and surface membranes.

### Resource Availability

#### Lead Contact

Further information and requests for resources and reagents should be directed to and will be fulfilled by the Lead Contact, Etienne Pays (epays@ulb.ac.be).

#### Materials Availability

All unique/stable reagents generated in this study are available from the Lead Contact with a completed Materials Transfer Agreement. However, we only have a very limited amount of anti-VSG IgMs.

#### Data and Code Availability

The published article includes all proteomic data generated or analyzed during this study.

## Methods

All methods can be found in the accompanying Transparent Methods supplemental file.

## Supplemental Information

Document S1. Transparent Methods and Figures S1–S7

Table S1. Proteome Associated with TbKIFC1, Related to Figure 2 Mass spectrometry analysis of *Tb*KIFC1-BL proteins bound to streptavidin in extracts from WT and VHS7 *Tb*KIFC1-BL addback cells. Biotinylated proteins are highlighted in yellow.
