# Supplementary material for: The Trypanosoma Brucei KIFC1 Kinesin Ensures the Fast Antibody Clearance Required for Parasite Infectivity
Source: iScience. 2020 Aug 20;23(9):101476. doi: 10.1016/j.isci.2020.101476 (PMC7479354; doi:10.1016/j.isci.2020.101476)
Supplement: Document S1. Transparent Methods and Figures S1–S7 [file mmc1.pdf]

iScience, Volume *23*

Supplemental Information

The *Trypanosoma Brucei* KIFC1 Kinesin Ensures the Fast Antibody Clearance Required for Parasite Infectivity

Laurence Lecordier, Sophie Uzureau, Gilles Vanwalleghem, Magali Deleu, Jean-Marc Crowet, Paul Barry, Barry Moran, Paul Voorheis, Andra-Cristina Dumitru, Yoshiki Yamaryo-Botté, Marc Dieu, Patricia Tebabi, Benoit Vanhollebeke, Laurence Lins, Cyrille Y. Botté, David Alsteens, Yves Dufrêne, David Pérez-Morga, Derek P. Nolan, and Etienne Pays

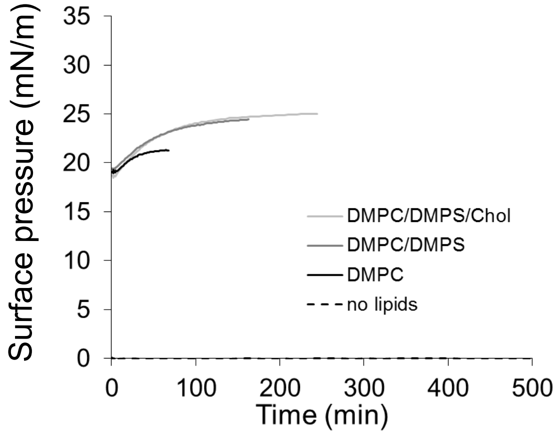

Figure S1. ***Tb*****KIFC1 interaction with synthetic membranes (related to Fig. 1)**

Adsorption kinetics of the mutant VHS7 domain into an air-water interface (without lipids) or into a lipid monolayer composed by DMPC or DMPC-DMPS (1:1) or DMPC-DMPS-Cholesterol (1:1:2).

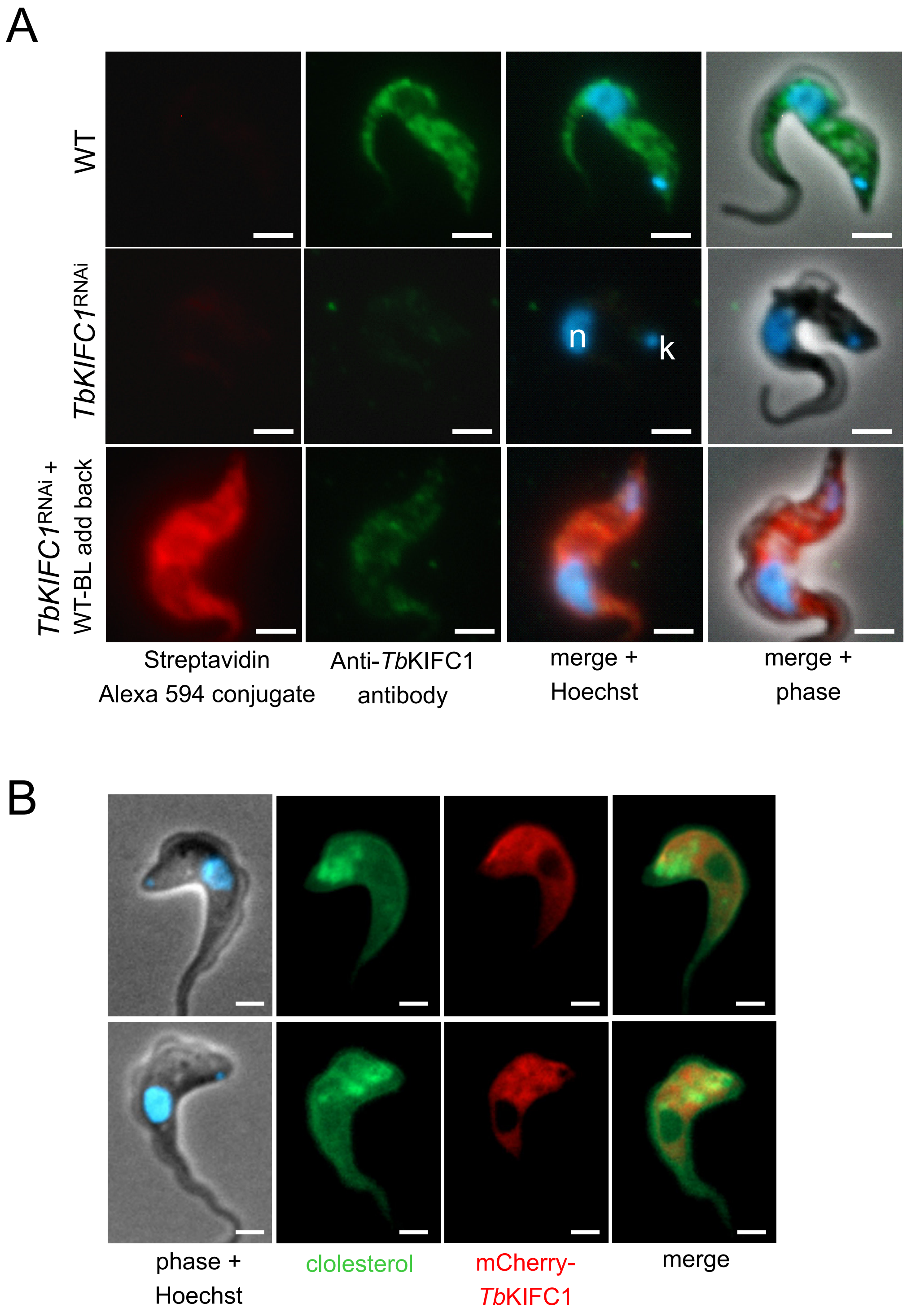

Figure S2. *Tb*KIFC1 expression (related to Fig. 1)

(A) Immunofluorescence analysis of *Tb*KIFC1 expression in WT and *TbKIFC1*^{RNAi} parasites, with or without addback expression of BL-WT *Tb*KIFC1. (n = nucleus; k = kinetoplast; bar = 2 µm)

(B) Localization of endogenously tagged mCherry-*Tb*KIFC1 in WT parasites labelled with Top Fluor cholesterol and Hoechst (bar = 2µm).

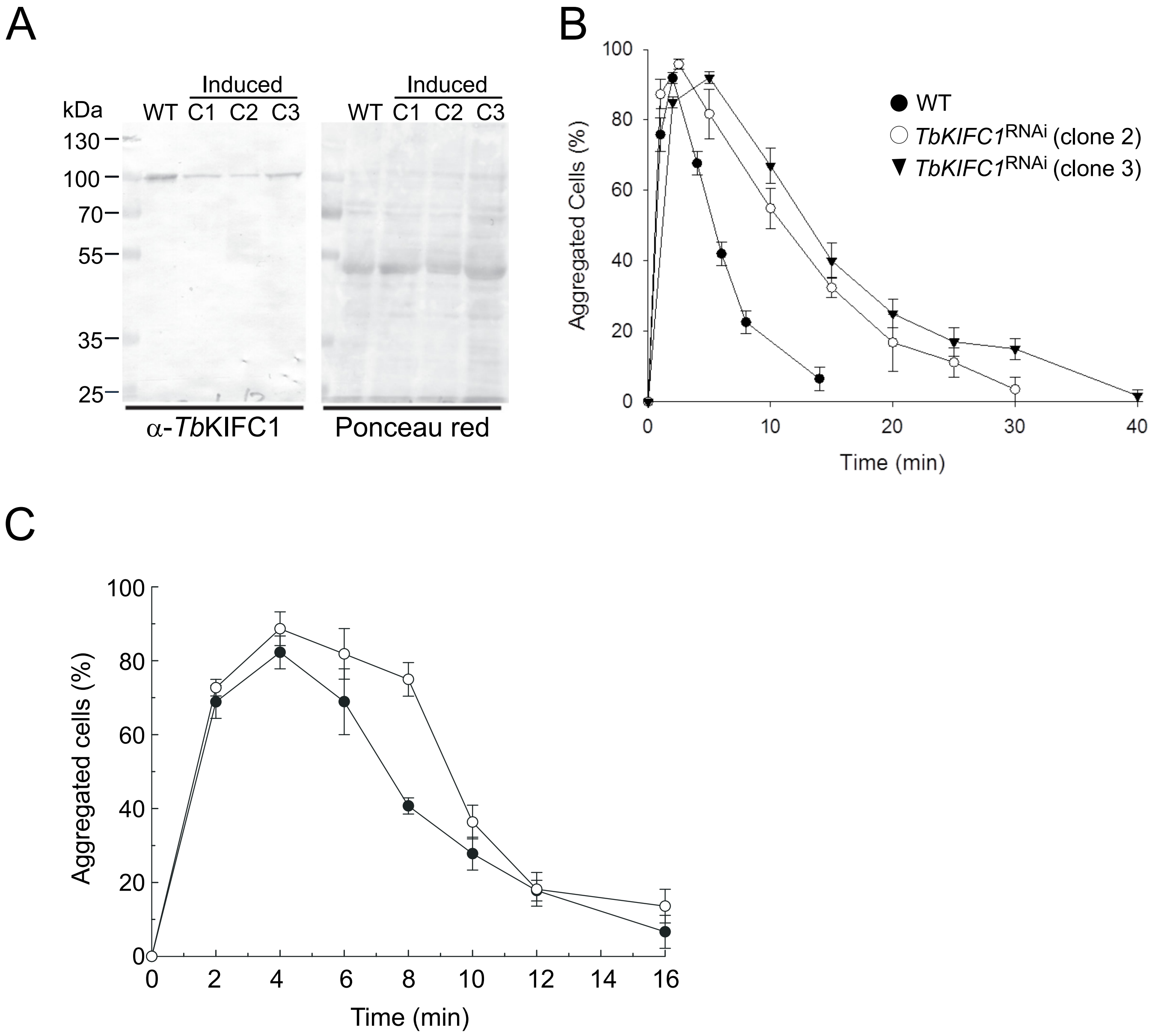

Figure S3. *Tb*KIFC1 levels in different cloned *TbKIFC1*^{RNAi} cell lines and kinetics of WT or *TbKIFC1*^{RNAi} parasite disaggregation following addition of anti-VSG IgGs or IgMs (related to Fig. 4)

(A) Western blot analysis of *Tb*KIFC1 levels (~ 100 kDa band) in WT and three different cloned *TbKIFC1*^{RNAi} cell lines (clones 1-3: C1, C2, C3) following 48 h of RNAi induction by doxycyclin.

(B) WT and two different *TbKIFC1*^{RNAi} cell lines (Clones 2 and 3 both 48 h induction by doxycyclin) were incubated with purified anti-VSG IgGs (1 µg/ml) and the clearance of surface-bound antibodies was monitored by a cell aggregation assay (error bars: S.D.; n=3).

(C) Kinetics of *TbKIFC1*^{RNAi} parasite disaggregation following addition of anti-VSG IgM. Two different *TbKIFC1*^{RNAi} cell lines, clones 2 (●) and 3 (○), both 48 h induction by doxycycline, were incubated with purified anti-VSG IgMs (4 µg/ml) and the clearance of surface-bound antibodies was monitored by a cell aggregation assay (error bars: S.D.; n=3).

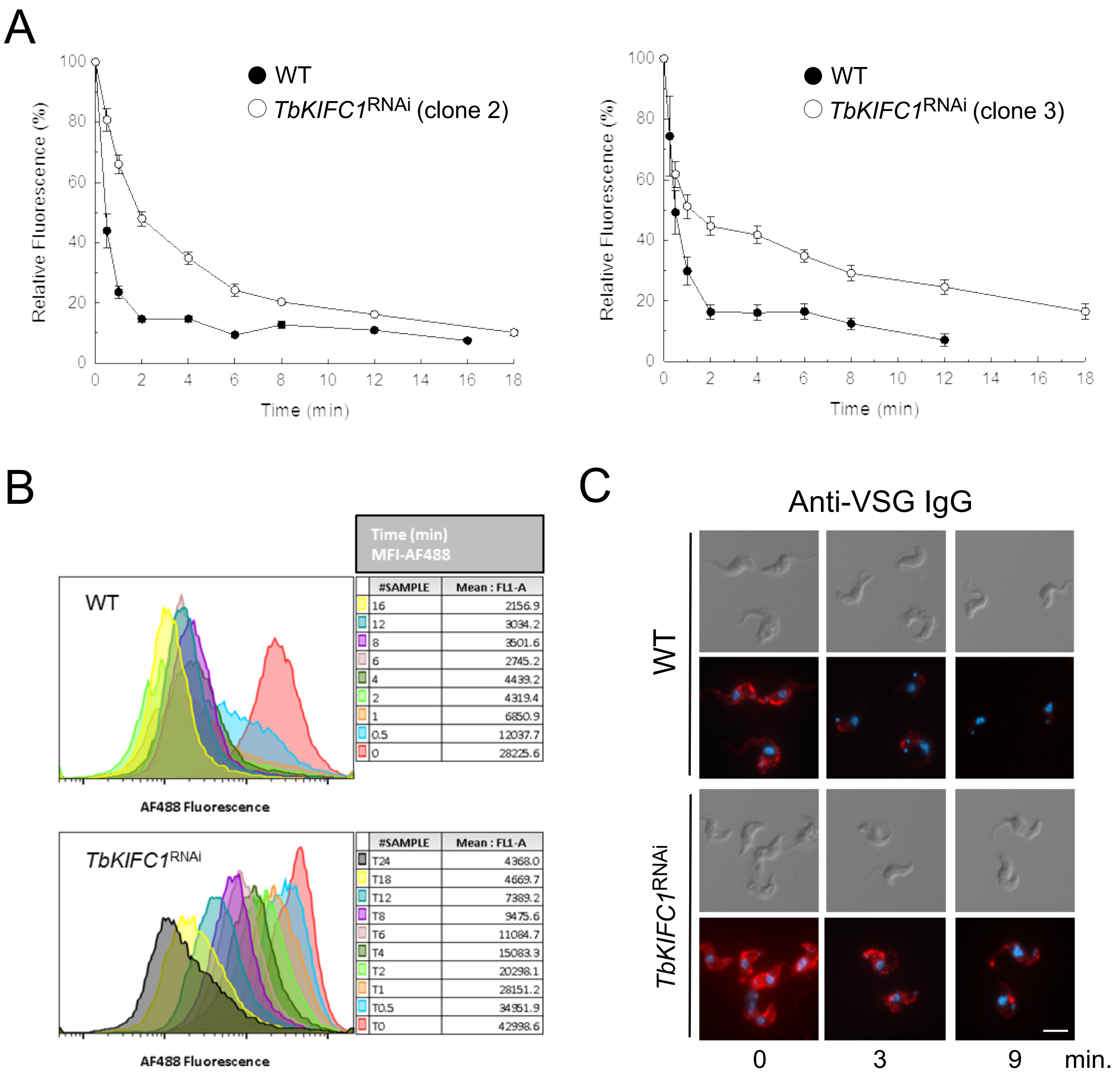

Figure S4. Clearance of VSG-IgG complexes from the surface of WT and *TbKIFC1*^{RNAi} cells (related to Fig. 4)

(A) Clearance of surface-bound antibodies (anti-VSG IgG) was determined by flow cytometry of WT and *TbKIFC1*^{RNAi} cells (clones 2 and 3 from Fig. S3, 48 h of RNAi induction by doxycyclin) incubated with anti-VSG immune serum. The data are expressed as % median fluorescence intensity detected relative to time zero (error bars: S.D.; n=3).

(B) WT and *TbKIFC1*^{RNAi} cells (clone 1, 48 h of RNAi induction by doxycyclin) were incubated with anti-VSG immune serum and the clearance of surface-bound antibodies was determined by flow cytometry. The panels show representative samples (data fromFig.3D) of individual fluorescence intensity distributions of surface Alex488 fluorescence *versus* cell count (total 10,000) at each time point.

(C) Fluorescence microscopy representative kinetics of clearance of anti MiTat 1.1 VSG IgGs from the surface of WT and *TbKIFC1*^{RNAi} (48 h induction) parasites. Cells were incubated for 30 min at 4°C with anti-VSG antibodies (IgG) and then incubated at 37°C for 0, 3 and 9 min. Surface-bound antibodies are revealed by immunofluorescence with Alexa 594 anti-rabbit secondary antibodies. bar = 5µm.

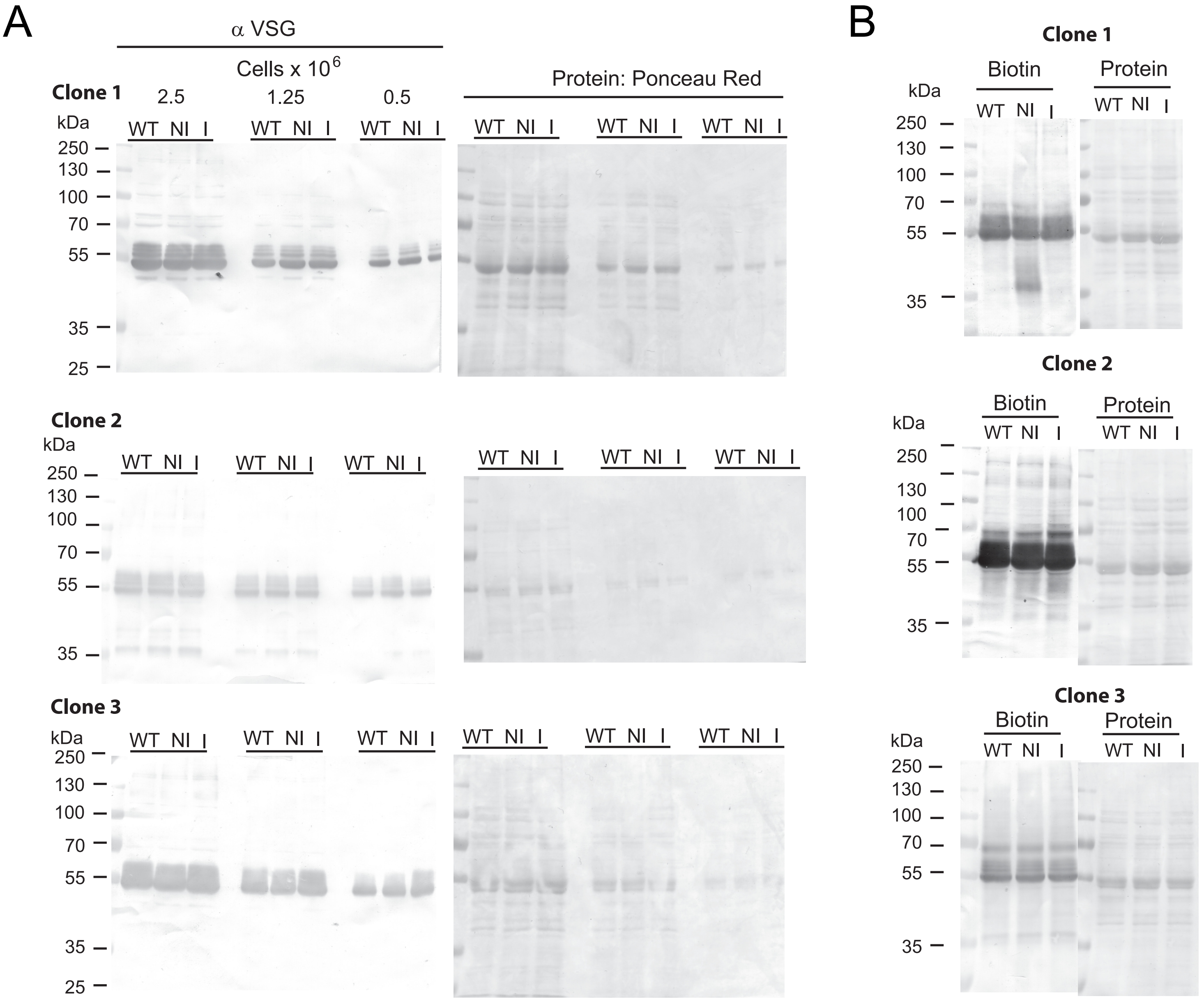

Figure S5. VSG titration and surface biotinylation in WT and *TbKIFC1*^{RNAi} cell lines (related to Fig. 4)

(A) Level of VSG expression, as determined by Western blotting using anti-MiTat 1.1VSG antibodies. Protein loading was assessed by Ponceau Red staining prior to blotting. Titrations were performed by loading various cell equivalents of WT, non-induced (NI) and induced (I) cells (48 h of RNAi induction by doxycyclin).

(B) Protein exposure on the parasite surface, as determined by surface biotinylation using non-cleavable EZ-link Sulfo-NHS-biotin. Biotinylated proteins were detected in samples (2.5 x 10^6 cells) of WT, non-induced (NI) and induced (I) *TbKIFC1*^{RNAi} cells (48 h of RNAi induction by doxycyclin) by streptavidin-AP blotting (left lanes in each panel). Protein loading was assessed by Ponceau Red staining prior to blotting (right lanes in each panel).

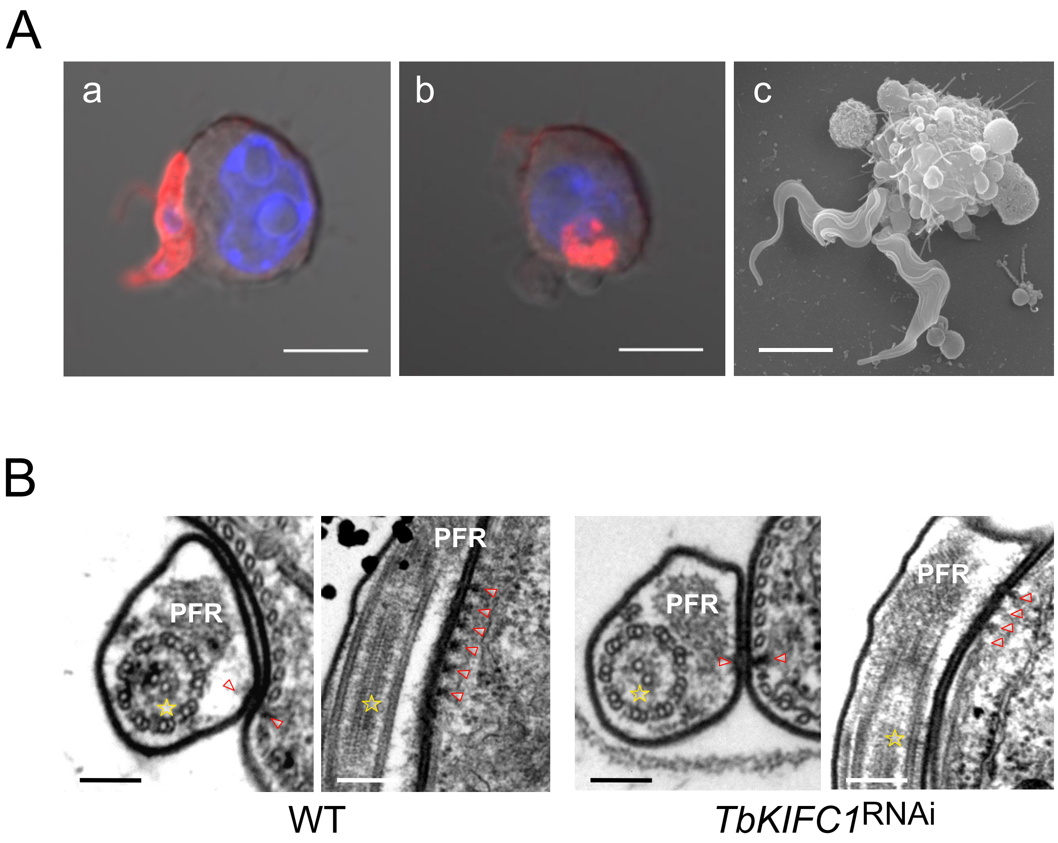

Figure S6. Phenotypic characteristics of *TbKIFC1*^{RNAi} parasites (related to Fig. 4)

(A) Uptake by macrophages. Representative IF and SEM images of macrophage-bound (a,c) or macrophage–phagocytosed (b) trypanosomes, after incubation for 30 min with anti-MiTat 1.1 VSG antibodies (red: anti-VSG antibodies; blue: nuclear DAPI staining; bar = 5µm).

(B) No alteration of the flagellum. TEM images of cross and longitudinal sections of the flagellum in WT and *TbKIFC1*^{RNAi} trypanosomes (star = axoneme; arrow point = FAZ filament; bar = 200 nm).

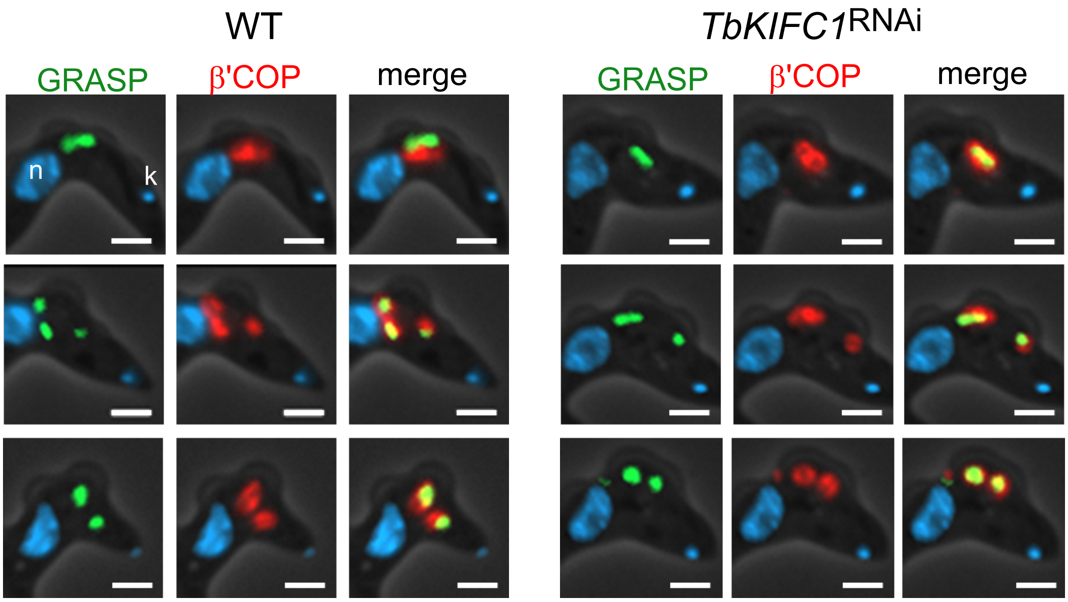

Figure S7. Association of β' COP with the Golgi (related to Fig. 6)

Fluorescence analysis of red mCherry-β'COP and green YFP-GRASP conjugates in WT and *TbKIFC1*RNAi parasites (blue = Hoechst staining of DNA in nucleus (n) and kinetoplast (k)). (Bar = 2 μm)

Transparent Methods

Ethics statement. This research was approved by the ethics committee of the Institute for Molecular Biology and Medicine (IBMM). All mice were housed in our specified pathogen-free facility and the experiments were performed in compliance with the relevant laws and institutional guidelines (license LA1500474).

Parasites. *Trypanosoma brucei brucei* parasites were grown in HMI9 supplemented with 10% foetal bovine serum, 10% Serum Plus at 37°C in 5% CO_2. The 328-114 *TbKIFC1*^{RNAi} cell line and add backs were described previously (Vanwalleghem et al., 2015). A new *TbKIFC1*^{RNAi} cell line was generated using the SmOx plasmid (Poon et al., 2012), with the p2T7-177 vector and a MITat 1.1 *T. brucei* clone as the parental line. This MITat1.1 cell line was used in experiments with anti-VSG antibodies; clone 1 was used in most experiments, and two additional clones were used in experiments shown in supplemental figures. RNAi was induced by incubation with 1 µg/ml doxycycline for 48 h prior to experiments.

Production of recombinant WT and mutant VHS domain. The sequence encoding the VHS domain of *Tb*KIFC1 (amino acids 1 to 156) was amplified by PCR from recoded WT or VHS7 mutant *TbKIFC1* and inserted in the pStaby1.2 vector (Delphi Genetics) in fusion with the Histidine tag. Expression was induced overnight at 30°C with 1 mM isopropyl β-D-thiogalactoside. Recombinant proteins were purified from inclusion bodies (Vanhamme et al., 2003).

Binding of recombinant VHS to lipid strips. Lipid binding assays were performed on lipid-coated strips (Wan et al., 2008).

Molecular modelling of *Tb*KIFC1. The *Tb*KIFC1 sequence was aligned with that of the VHS domains of TOM1 (PDB code: 1ELK) and of the human signal transducing adaptor molecule 2 (1X5B). The *Tb*KIFC1 model was build using the SWISS model (Arnold et al., 2006).

Interaction of *Tb*KIFC1 helices H7H8 with POPS or POPC. Interaction of H7H8 with POPS/POPC was first simulated by a docking method as previously described (Deleu et al., 2014; Lins et al., 1999). Briefly, the position of the peptide stays still while the lipid molecule moves along and around it (more than 10^6 rotations and translations). For each position, the energy of interaction between the two molecules is calculated as the sum of Van der Waals, electrostatic and hydrophobic terms. The energy values together with the coordinates of all assemblies are stored in a matrix and classified according to decreasing values. The most stable matching is used to decide of the position of the first lipid. The position of the second lipid is then defined as the next most energetically favourable orientation stored in the hypermatrix taking steric and energetic constraints due to the presence of the first lipid molecule into account. For the next lipid molecule, the same process is repeated but the positions of all surrounding molecules are modified alternatively in order to find the lowest energy state. Five molecules of lipids (POPS or POPC) were docked around the peptide and the average interaction energy was calculated. To have a more realistic model, the interaction of *Tb*KIFC1 H7H8 with a POPC/POPS membrane was simulated by molecular dynamics. The peptide was first modeled in an atomistic representation. The martinize script (De Jong et al., 2013) was used to convert the structure to a coarse grained (CG) representation and to generate the topology for the MARTINI force field (Marrink et al., 2007; Monticelli et al., 2008). The CG peptide is placed in a simulation box with a POPC/POPS membrane (9:1

molar ratio) with the insane script (Ingolfsson et al., 2014). A 5000 steps steepest-descent energy minimization is performed to remove any steric clashes and a 1 μs production simulation is run. Temperature and pressure are coupled at 300 K and 1 bar using the weak coupling Berendsen algorithm (Berendsen et al., 1984) with $\tau T = 1$ ps and $\tau P = 1$ ps. Pressure is coupled semi-isotropically. Non bonded interactions are computed up to 1.2 nm with the shift method. Electrostatics are treated with $\varepsilon = 15$ and compressibility is 10^5 (1/bar). CG simulations have been carried out by using Gromacs 4.5.4 (Hess et al., 2008). At the end of simulation, the system is transformed to an atomistic resolution with the reverse script (Wassenaar et al., 2014) and all atom MD is further performed with Gromos G54a7 force field (Schmid et al., 2011). All the systems studied are first minimized by steepest descent for 5000 steps. A 100 ps simulation is run with the peptide under position restraints and NVT conditions, followed by a 1 ns simulation with the peptide under position restraints and NPT conditions. Production simulations of 250 ns are performed. Periodic boundary conditions (PBC) are used with a 2 fs time step. All the systems are solvated with SPC water (Hermans et al., 1984) and MD runs are carried out in NPT conditions (298 K and 1 bar). Temperature is maintained by using the v-rescale method (Bussi et al., 2007) with $\tau T = 0.2$ ps and isotropic pressure by using Berendsen barostat (Berendsen et al., 1984), with a compressibility of 10^5 (1/bar) and $\tau P = 1$ ps. Electrostatic interactions are treated by using the particle mesh Ewald (PME) method (Essmann et al., 1995) van der Waals and electrostatics are treated with a 1.0 nm cut-off. Bond lengths are maintained with the LINCS algorithm (Hess et al., 1997). Trajectories were analyzed with GROMACS 4.5.4 tools as well as with homemade scripts and softwares. 3D structures were analyzed with PyMOL (Schrödinger, 2010) and VMD (Humphrey et al., 1996) softwares.

***Tb*KIFC1 VHS, VHS7 and H7H8 adsorption on lipid monolayers.** Adsorption experiments of either recombinant *Tb*KIFC1 VHS and VHS7 domain or synthetic H7H8 peptide to air-aqueous medium interface in the absence or presence of DMPC, DMPC-DMPS (1:1) or DMPC-DMPS-cholesterol (1:1:2) membranes were performed in a KSV Minitrough (9.5 cm x 21 cm) equipped with a Wilhelmy plate. The sub-phase was 10 mM Tris-HCl (pH 7.4) and temperature was maintained at 22 ± 1°C during all experiments. The sub-phase was continuously stirred. Lipids were spread at the air-water interface to reach the desired initial surface pressure (Π_i ~18.5 mN/m). 30 min-waiting was required for solvent evaporation and film stabilization. *Tb*KIFC1 VHS or H7H8, dissolved in DMSO at $12.4.10^{-5}$ M, was then injected (20 µL) underneath the pre-formed lipid monolayer (final concentration in sub-phase: $3.09.10^{-8}$ mol/L and 0.59×10^{-6} mol/L for VHS and H7H8 respectively) and its adsorption was followed by tensiometry as an increase of surface pressure (Nasir et al., 2017). No change of the surface pressure was observed after injection of DMSO. The attractiveness factor is defined as $(\Delta\Pi_0 - \Delta\Pi_x)/\Delta\Pi_x$ with $\Delta\Pi_0$ corresponding to the y-intercept of the linear regression of $\Delta\Pi_{eq}$ vs Π_i, and $\Delta\Pi_x$, which is the equilibrium surface pressure increase without lipid monolayer. The uncertainties of the maximal insertion pressures (MIP) and of the attractiveness factor were calculated using IgoPro Software.

Construction of Biotin Ligase (BL)-conjugated *Tb*KIFC1. The Biotin Ligase (BL) gene (BirA*) gene was inserted by recombination in the recoded addback versions of WT and VHS7 *TbKIFC1* genes in order to fuse the BL domain between amino acids 379 and 380 of *Tb*KIFC1, within a poly-proline stretch. The two constructs were transfected in *TbKIFC1*^{RNAi} trypanosomes that were selected for hygromycin resistance. Resistant parasites were analysed for expression and functionality of the *Tb*KIFC1-BL fusion protein by *TbKIFC1* qRT-PCR and lysis test with APOL1. Biotinylation activity of the fusion protein was verified after 24h

incubation with 1 µg/ml doxycycline and 50 µM Biotin, by Western blot revealed with Streptavidin-peroxydase and chemioluminescence, and by immunofluorescence with streptavidin-Alexa-594.

Mass spectrometry analysis of *Tb*KIFC1-BL proteins bound to streptavidin. WT parasites, WT and VHS7 *Tb*KIFC1-BL addback cells were incubated for 24 h with 1 µg/ml doxycycline before addition of 50 µM biotin for further 24 h. Parasites were lysed in phosphate-buffered saline (PBS) containing 1% NP40, 0.25% Na-deoxycholate and 0.2% SDS for 1 h on ice, and the lysates were cleared by centrifugation at 21,000 g. The supernatants were incubated for 2 h at 4°C with streptavidin agarose beads (Pierce). The beads were washed three times with lysis buffer and twice with PBS buffer. Proteins were eluted from beads by boiling for 5 min in Laemmli sample buffer containing 1 mM biotin, and separated by SDS PAGE. Each lane was analysed by mass spectrometry after tryptic digestion. The peptides were analyzed using nano-LC-ESI-MS/MS maXis Impact UHR-TOF (Bruker, Bremen, Germany) coupled with a UPLC Dionex UltiMate 3000 (Thermo). The lanes were excised from SDS PAGE gels, and the proteins were digested with trypsin by in-gel digestion. The gel pieces were washed twice with distilled water and then shrunk with 100 % acetonitrile. Proteolytic digestion was performed by the addition of 6 µl of modified trypsin (Promega) suspended in 50 mM NH_4HCO_3 cold buffer. Proteolysis was performed overnight at 37°C. The supernatants were collected and the eluates were kept at – 20°C prior to analysis. The digests were separated by reverse-phase liquid chromatography using a 75 µm X 250 mm reverse phase Thermo column (Acclaim PepMap 100 C18) in an Ultimate 3000 liquid chromatography system. Mobile phase A was 95 % of 0.1 % formic acid in water and 5 % acetonitrile. Mobile phase B was 0.1 % formic acid in acetonitrile. The digests (15 µl) were injected, and the organic content of the mobile phase was increased linearly from 4 % B to 35

% in 35 min and from 35 % B to 90 % B in 5min. The column effluent was connected to a Captive Spray (Bruker). In survey scan, MS spectra were acquired for 0.5 s in the m/z range between 50 and 2200. The 10 most intense peptides ions 2^+ or 3^+ were sequenced. The collision-induced dissociation (CID) energy was automatically set according to mass to charge (m/z) ratio and charge state of the precursor ion. MaXis and Thermo systems were piloted by Compass HyStar 3.2 (Bruker). Peak lists were created using DataAnalysis 4.1 (Bruker) and saved as MGF file for use with ProteinScape 3.1 (Bruker) with Mascot 2.4 as search engine (Matrix Science). Enzyme specificity was set to trypsin, and the maximum number of missed cleavages per peptide was set at one. Carbamidomethylation was allowed as fixed modification, oxidation of methionine and biotinylation (K) were allowed as variable modification. Mass tolerance for monoisotopic peptide window was 7 ppm and MS/MS tolerance window was set to 0.05 Da. The peak lists were searched against the *Trypanosoma* taxonomy from UNIREF 100 (176,332 sequences) and proteins identified in the WT control (without BL) were subtracted from the results. Relative enrichment of proteins found only in the two *TbKIFC1*-BL strains was analyzed in TriTryp database for Gene Ontology (GO) term enrichment.

Production of rAPOL1. Recombinant APOL1 was prepared according to the procedure already described (Vanhamme et al., 2003). The purity and concentration were verified by SDS-PAGE and Coomassie blue staining.

***In vitro* trypanolysis and growth assays.** Lysis assays were performed as in (Pérez-Morga et al., 2005; Vanhamme et al., 2003). Normalizations were performed to untreated controls. For the measurements of SHP1 peptide toxicity, parasites were incubated for 90 min at 37°C with the peptide before counting.

Mice infection. NMRI and C57BL/6 mice, either WT or µMT KO, were used for these experiments. 24 h before mice infection, the *TbKIFC1*^{RNAi} and addback parasites were incubated *in vitro* with 1µg/ml doxycyclin to induce RNAi, and mice were given 1 mg/ml doxycyclin with 5g/L sucrose in drinking water (Lecordier et al., 2005). For infection, parasites were concentrated to inject intraperitoneally 10^5 cells in 100 µl per mouse. Drinking water was replaced the day of infection and every two days during the course of infection. Parasitaemia was monitored by tail blood puncture. WT and µMT KO C57BL/6 mice were infected under the same conditions. Sample size was empirically estimated and validated by the stability of the standard deviation of the growth curves. Within each experiment, animals used were morphologically undistinguishable (same strain, age and weight) and allocated randomly to each group. No blinding was done. For experiments aiming at evaluating the effects of phagocytic cells, mice were injected 3 days before infection with 200µl of liposomes containing either phosphate buffered saline (PBS) or clodronate (Liposoma Research).

Antibodies. Rabbit immune serum and purified anti- MITat 1.1 VSG antibodies (IgMs and IgGs) were generated as previously described (O'Beirne et al., 1998). Normal mouse serum was obtained from healthy mice after clotting for 1 h at 37°C.

Aggregation assay. This assay was performed essentially as in O'Beirne et al., (1998). Monomorphic MITat 1.1 bloodstream forms were incubated at 37 °C (5% CO_2) at 1 x 10^7 cells/ml in HMI9 containing 10% fetal calf serum. After preincubation for 10 min, purified anti-VSG IgG or IgM was added. Samples (10 µl) were removed at various times (see Figs. 4, S3, S4) and diluted in 90 µl of ice-cold PSG [3 mM NaH_2PO_4, 57 mM Na_2HPO_4, 44 mM

NaCl, 5 mM KCl, 1 mM $MgCl_2$, 10 mM glucose and 70 mM sucrose (pH 8.0)] and the numbers of free non-aggregated cells were counted in duplicate immediately. The percentage of aggregated cells was determined (O'Beirne et al., 1998).

Analysis of clearance of surface bound antibodies by flow cytometry. The clearance of surface bound anti-VSG antibodies from individual cells was also monitored by flow cytometry. Cultures of exponentially growing MITat 1.1bloodstream forms were collected by centrifugation for 5 min at 1500 g, and resuspended (10^7 cells/ml) in ice-cold HMI9 + fetal calf serum. The suspension of cells (5 ml) was incubated on ice with rabbit immune serum (1:1000 dilution) for 1 h and then transferred to 10 ml of ice-cold HMI9 + fetal calf serum, and centrifuged for 5 min at 1500 g (4 °C). The supernatants were removed and the pellets were quickly resuspended in 5 ml of pre-warmed (37 °C) HMI9 medium. A sample (500 µl) was removed immediately and transferred to an equal volume cold fixation solution (6% PFA in PBS, pH 7.5) in a 15 ml centrifuge tube and kept on ice. At various times additional samples (see Figs. 4 and S5) were removed and transferred to fixation solution as above. After 15 min fixation 14 ml of cold quench solution (PBS + 2 mM glycine) were added and the cells were centrifuged for 5 min at 1500 g (4 °C), washed once in PBS and resuspended in 250 µl (~ 2 x 10^7 cells/ml) in cold PBS. Samples (0.1 ml) of fixed cells were incubated for 1 h at 4 °C with an equal volume of Alexa-488 labelled goat anti-rabbit IgG diluted 1/800 in PBS containing 1% fetal calf serum and 1% BSA. The cells were centrifuged for 30 s at 10,000 g and resuspended in 200 µl of PBS before analysis for Alexa-488 fluorescence by flow cytometry. Flow cytometry was performed on the Accuri C6 flow cytometer (BD; San Jose, USA); AlexaFluor488 fluorescence was detected through the 488 nm laser-533/30 nm filter set.

Analysis of the internalization of surface biotinylated proteins. Endocytosis of surface proteins was monitored using a previously described surface biotinylation assay (Engstler et al., 2004). Cultures of MITat 1.1 cells (0.5-1 x 10^6 cells/ml) were chilled on ice and collected by centrifugation for 5 min at 1500 g (4 °C), and resuspended in ice-cold PSG. Biotinylation was performed on ice by incubating the cells (10^8/ml) with EZ-Link Sulfo-NHS-SS-Biotin (1.6 mM) for 10 min. The cells were transferred to 19 volumes of ice-cold PSG supplemented with 10 mM Tris (pH 8) and centrifuged for 5 min at 1500 g (4 °C), washed and resuspended in PSG (10^8 cells/ml) and transferred to 9 volumes of pre-warmed HMI9 +10% fetal calf serum, and incubated at 37 °C. At various times (see Fig. 4), 500 µl samples were removed and transferred to 19 volumes of ice-cold PSG + 1% BSA, centrifuged for 5 min at 1500 g (4 °C), washed and resuspended in PSG (10^7 cells/ml). An equal volume of stripping buffer [PSG + 10% fetal calf serum and 50 mM reduced glutathione (pH 8.5)] was added and the cells were incubated on ice for 15 min. Ten volumes of ice-cold PSG were added and the cells were collected by centrifugation as before and washed with cold PSG, resuspended in PSG at 5 x 10^7 cells/ml and fixed by the addition of an equal volume cold fixation solution (6% PFA in PBS, pH 7.5) in a 15 ml centrifuge tube and kept on ice. After 15 min fixation 14 ml of cold quench solution (PBS + 2 mM glycine) were added and the cells were centrifuged for 5 min at 1500 g (4 °C), washed once in PBS and resuspended in 250 µl (~ 2 x 10^7 cells/ml) in cold PBS. Samples (0.1 ml) of fixed cells were incubated for 1 h at 4 °C with an equal volume of 40 µg/ml phycoerythrin (PE)-labelled streptavidin in PBS containing 1% BSA and 0.25% Triton X-100. The cells were centrifuged for 30 s at 10,000 g and resuspended in 200 µl of PBS before analysis by flow cytometry. PE fluorescence was detected using the 488 nm laser 585/40 nm filter set.

Return of biotinylated proteins to the surface. Surface-biotinylated bloodstream form cells (as above) were transferred to 9 volumes of pre-warmed HMI9 + 10% fetal calf serum and incubated at 37 °C for 15 min. The cell suspension was then transferred to 19 volumes of ice-cold PSG, centrifuged for 5 min at 1500 g (4 °C), washed and resuspended in PSG (10^7 cells/ml). An equal volume of stripping buffer (ice-cold PSG + 10% fetal calf serum), glutathione was added and the cells were incubated on ice for 15 min. Ten volumes of ice-cold PSG were added and the cells were collected by centrifugation as before and washed with cold PSG. The cells were then resuspended (10^7 cells/ml) in pre-warmed HMI9 + 10% fetal calf serum and incubated at 37 °C. At various times (see Fig. 4), 500 µl samples were removed and added to an equal volume of cold fixation solution (6% PFA in PBS, pH 7.5). After fixation and washing, the cells were resuspended in 250 µl (~ 2 x 10^7 cells/ml) in cold PBS. Surface fluorescence was detected by incubation of fixed cells for 1 h at 4 °C with an equal volume of 40 µg/ml PE-labelled streptavidin in PBS containing 1% BSA. The cells were centrifuged for 30 s at 10,000 g and resuspended in 200 µl of PBS before analysis by flow cytometry. PE fluorescence was detected using the 488 nm laser 585/40 nm filter set.

FRAP analysis. FRAP assays were performed essentially as in Uzureau et al. (2013). Briefly, 2 x 10^6 bloodstream *T. brucei* cells were labelled with 1 µM sulfo-N-hydroxysulfosuccinimide coupled Atto 488 fluorescent dye (ATTO-TEC GmbH, Siegen) for 15 min on ice and washed two times with cold PBS buffer containing 80 mM glucose. In some experiments, WT parasites were preincubated for 30 min at 37°C with 2.5 mM MBCD before labeling. The trypanosomes were immobilized on a 1.25 % low melting point agarose pad sealed with rubber glue. The samples were put in an incubation chamber thermo-stabilized at 30 °C. Under these conditions the parasites remained alive (flagellum beating) for at least 6 h. FRAP analysis was performed with a Zeiss LSM 710 confocal microscope.

For each cell 20 pre-bleach and 80 post-bleach acquisitions were made. The mobile fraction was calculated according to the Zeiss Zen 2010 software using a double normalization (background and reference image).

Mobility assay. MITat 1.1 WT and *TbKIFC1*^{RNAi} parasites were concentrated at 2.5 x 10^6 cells/ml and maintained for 30 min at 37°C with or without anti-MITat 1.1 VSG antibody. The parasites were then loaded in microwells containing solidified Matrigel. Images were acquired for 40 sec at 500 ms interval (Axio Observer Z1). Cell tracking was performed using Fiji software and the MTrackJ module (Meijering et al., 2012).

Phagocytosis assay. Phagocytosis assay of MITat1.1 WT and *TbKIFC1*^{RNAi} trypanosomes was performed with murine RAW264.7 macrophages seeded on coverslips exactly as in Cheung et al. (2016). Parasites were opsonized with 10% normal mouse serum or anti-MITat 1.1 antibodies for 30 min at 37°C before co-incubation with activated macrophages for 90 min. Cells were fixed with 2% PFA and permeabilized in methanol at -20°C for 30 s. Surface-bound and internalized parasites were detected with anti-MITat 1.1 VSG and anti-rabbit Alexa Fluor 594 antibodies. Coverslips were mounted before immunofluorescence analysis. Consecutive fields were acquired and parasites were counted for 500 to 1,000 macrophages in each condition.

SEM analysis. Parasites and macrophages seeded on coverslips were co-incubated as described above and then fixed overnight at 4°C in 2.5% glutaraldehyde, 0.1M cacodylate buffer (pH 7.2), and post-fixed in OsO4 (2%) in the same buffer. After serial dehydration in increasing concentrations of ethanol, samples were dried at critical point and coated with platinum by standard procedures. Observations were made in a Tecnai FEG ESEM QUANTA

200 (FEI) and images capture with a secondary electron detector and processed by SIS iTEM (Olympus) software.

TEM analysis. Cells were fixed for 1 h at room temperature in 2.5% glutaraldehyde in culture medium, and postfixed in 2% OsO4. After serial dehydration in increasing ethanol concentrations, samples were embedded in agar 100 (Agar Scientific Ltd., UK) and left to polymerize for 2 days at 60 °C. Ultrathin sections (50–70-nm thick) were collected in Formvar-carbon- coated copper grids by using a Leica EM UC6 ultramicrotome and stained with uranyl acetate and lead citrate. Observations were made on a Tecnai10 transmission electron microscope (FEI), and images were captured with an Olympus VELETA camera and processed with AnalySIS and Adobe Photoshop softwares.

Atomic force microscopy. *T. brucei* parasites were fixed for in 5 min at 4°C (on ice) in 3.5% HCHO while in culture medium at 37°C. Samples were washed 3 times for 5 min and resuspended in 0.1 M sodium cacodylate buffer (pH 7.4). Cells were then immobilized onto poly-L-lysine-coated glass-bottomed Petri dishes (WillCo). Dishes were first filled with a 0.5 mg/ml poly-L-lysine solution (PLL, Mw ~ 70-150 kDa) in PBS, incubated at 37°C for 30 min, washed with buffer for 3 min and dried in a laminar flow hood for 2 hours at room temperature. Either WT or *TbKIFC1*^{RNAi} parasite suspensions were plated onto the PLL-coated dishes overnight. The dishes were then washed 3 times with PBS to remove non-attached cells. AFM experiments were performed with a Bioscope Resolve AFM (Bruker) operated in PeakForce QNM mode at ~25-30°C in PBS. PeakForce QNM Live Cell probes (Bruker) with spring constants ranging from 0.08 N to 0.1 N/m and a tip radius of curvature of ∼65 nm were used for the measurements. Spring constants were calibrated by the manufacturer with a vibrometer (OFV-551, Polytec, Waldbronn) and they were used to

determine the deflection sensitivity using the thermal noise method (Butt and Jaschke, 1995) before each experiment. FD-based multiparametric maps were acquired using a force setpoint of 1 nN while oscillating the AFM cantilever at 1 kHz with peak-to-peak oscillation amplitudes of 1 μm. Images were recorded using a scan rate of 0.2-0.6 Hz and 128 pixels per line. FD-based AFM maps were processed offline using the NanoScope Analysis 1.80 Software (Bruker). To extract the Young's modulus, we analyzed the retraction part of raw force-distance curves to avoid plastic deformation contributions. The best quality of the fit was obtained when by fitting the contact part of the curve with the Hertz model (Schillers et al., 2017)

$$F^{2/3} = \left(\frac{4}{3}\frac{E}{(1-\nu^2)}\sqrt{R}\right)^{2/3}\delta$$

where E is the Young's modulus, δ is the indentation depth, ν is the Poisson ratio, and R is the contact radius. We used a Poisson's ratio value of 0.3. The upper and lower boundaries of the fit region are defined as the percentage difference between the maximum and the minimum force. Therefore, these boundaries define the indentation depth taken into account in the analysis of the FD curves. We used fit ranges of 5-25%, which would mainly correspond to the contribution of the plasma membrane and 30-90%, which would comprise the contribution of the underlying microtubules (Dumitru et al., 2018).

Lipid analysis. Lipids were analyzed as previously described (Amiar et al., 2016; Dubois et al., 2018). Specifically, total lipid spiked with 10 nmol C13:0 fatty acid and PC (C21:0/C21:0) was extracted by chloroform:methanol, 2:1 (v/v) followed by biphasic separation by adding 0.2% KCl. An aliquot of resulted organic phase containing cholesterol was dried and analyzed by gas chromatography-mass spectrometry (Agilent 5977A-7890B) after derivatization of cholesterol to cholesterol-TMS by BSTFA-TMCS, 99:1 (Sigma). Phospholipids were separated by HPTLC with solvent system (hexane/diethylether/formic

acid, 80:20:2, v/v/v). The phospholipid spot was extracted for the quantification of fatty acids by gas chromatography-mass spectrometry (Agilent 5977A-7890B) after methanolysis. Fatty acid methyl esters and cholesterol-TMS were identified by their mass spectrum and retention time, and quantified by calibration curve generated with internal standards. Then cholesterol and phospholipid content was normalized according to the parasite cell numbers and C13:0 or C21:0 internal standard (Avanti Polar Lipids).

***In situ* tagging**. The pPOTv7 plasmid (Dean et al., 2015) was modified to replace the YFP gene by the mCherry coding sequence. For C-terminal tagging of GRASP (Tb927.11.2660), Sec15 (Tb927.11.7120), Sec1 (Tb927.9.1970), β'COP (Tb927.2.6050), FAZ1 (Tb927.4.3740), and N-terminal tagging of Rab11 (Tb927.8.4330), PCR primers described in TrypTag were used. PCR products were transfected by standard methods in the WT and *TbKIFC1*^{RNAi} cell lines. Parasites were selected with 5µg/ml blasticidin. For double tagging, the β'COP-mCherry tagged strains were transfected with a PCR product for YFP tagging of GRASP obtained from a version of pPOTv7 in which the blasticidin resistance gene had been replaced by the puromycin resistance gene.

Top Fluor cholesterol localization. Parasites were washed in HMI9 medium without serum and resuspended in HMI9 supplemented with 1% FCS and 5 µM Top Fluor cholesterol (Avanti Polar Lipids). Parasites were incubated for 50 min at 37°C before addition of Hoechst and a further 10 min incubation. Parasites were cooled down on ice, washed twice with cold HMI9 medium and resuspended in 10 µl before loading on agarose pads freshly prepared with HMI9 containing 10% FCS and 1.25% low melting point agarose. Cells were imaged immediately with Zeiss LSM 710 confocal or Axioimager M2 epifluorescence microscope with same parameters for all experiments. Fluorescence signals were analyzed with Fiji

software. To measure the intensity of the cholesterol signal within specific organelles, the mean fluorescence intensity of Top Fluor was measured within ROIs (regions of interest) that were delineated using thresholding of the mCherry signal corresponding to the different markers and after background subtraction. In addition, cholesterol enrichment in the membrane structure associated with FAZ was quantified by measuring the fluorescence intensity along a 2 pixels width line manually drawn based on the morphology of the Top Fluor signal. In order to control for variability in fluorescence signal, this quantification was done in all individual cells for each cell line and condition.

Cholesterol depletion. Depletion of cholesterol with methyl-β-cyclodextrin was obtained by a previous incubation of parasites at 37°C for 30 min and a wash with HMI9 medium. 10^5 cells were analyzed by flow cytometry (BD FACS Canto II).

Statistical analysis. Statistical analysis was performed using Prism software (GraphPad). Immunofluorescence data were obtained from randomly selected cells from three independent experiments, and the images shown are representative of the majority of cells. Quantitative data were represented as means ± standard deviation (SD); no sample was excluded. Normality of the data was analysed with the Shapiro-Wilk test. p-values were calculated by the Student's t test and one-way ANOVA (post hoc Dunnett's test) for multiple and single comparisons of normally distributed data respectively, and by the Mann-Whitney test for multiple comparisons of non-normally distributed data (*P < 0.05; **P < 0.01; ***P < 0.001; ****P <0.0001).

Supplemental References

Amiar, S., MacRae, J.I., Callahan, D.L., Dubois, D., van Dooren, G.G., Shears, M.J., Cesbron-Delauw, M.F., Maréchal, E., McConville, M.J., McFadden, G.I., et al. (2016) Apicoplast-localized lysophosphatidic acid precursor assembly is required for bulk phospholipid synthesis in *Toxoplasma gondii* and relies on an algal/plant-like glycerol 3-phosphate acyltransferase. PLoS Pathog. *12*, e1005765.

Berendsen, H. J. C., Postma, J. P. M., van Gunsteren, W. F., DiNola, A., and Haak, J. R. (1984) Molecular dynamics with coupling to an external bath. J. Chem. Phys. *81*, 3684.

Bussi, G., Donadio, D., and Parrinello, M. (2007) Canonical sampling through velocity rescaling. J. Chem. Phys. *126*, 014101.

Butt, H. J., and Jaschke, M. (1995) Calculation of thermal noise in atomic force microscopy. Nanotechnology *6*, 1.

Dean, S., Sunter, J., Wheeler, R.J., Hodkinson, I., Gluenz, E., and Gull, K. (2015) A toolkit enabling efficient, scalable and reproducible gene tagging in trypanosomatids. Open Biol. *5*, 140197.

De Jong, D. H., Singh, G., Bennett, W.F., Arnarez, C., Wassenaar, T.A., Schäfer, L.V., Periole, X., Tieleman, D.P., and Marrink, S.J. (2013) Improved Parameters for the Martini Coarse-Grained Protein Force Field. J. Chem. Theory Comput. *9*, 687–697.

Deleu, M., Crowet, J.M., Nasir, M.N., and Lins, L. (2014) Complementary biophysical tools to investigate lipid specificity in the interaction between bioactive molecules and the plasma membrane: A review. Biochim. Biophys. Acta *1838*, 3171-3190.

Dubois, D., Fernandes, S., Amiar, S., Dass, S., Katris, N.J., Botté, C.Y., and Yamaryo-Botté, Y. (2018) *Toxoplasma gondii* acetyl-CoA synthetase is involved in fatty acid elongation (of long fatty acid chains) during tachyzoite life stages. J. Lipid Res. *59*, 994-1004.

Dumitru, A. C., Poncin, M.A., Conrard, L., Dufrêne, Y.F., Tyteca, D., and Alsteens, D. (2018) Nanoscale membrane architecture of healthy and pathological red blood cells. Nanoscale Horizons *3*, 293-304.

Essmann, U., Perera, L., Berkowitz, M.L., Darden, T., Lee, H., and Pedersen, L.G. (1995) A smooth particle mesh Ewald method. J. Chem. Phys. *103*, 8577.

Hermans, J., Berendsen, H.J.C., Van Gunsteren, W.F., and Postma, J.P.M. (1984) A consistent empirical potential for water-protein interactions. Biopolymers *23*, 1513–1518.

Hess, B., Bekker, H., Berendsen, H.J.C., and Fraaije, J.G.E.M. (1997) LINCS: A linear constraint solver for molecular simulations. J. Comput. Chem. *18*, 1463–1472.

Hess, B., Kutzner, C., van der Spoel, D., and Lindahl, E. (2008) GROMACS 4: algorithms for highly efficient, load-balanced, and scalable molecular simulation. J. Chem. Theory Comput. *4*, 435–447.

Humphrey, W., Dalke, A., and Schulten, K. (1996) VMD: visual molecular dynamics. J. Mol. Graph. *14*, 33–38.

Ingólfsson, H.I., Melo, M.N., van Eerden, F.J., Arnarez, C., Lopez, C.A., Wassenaar, T.A., Periole, X., de Vries, A.H., Tieleman, D.P., and Marrink, S.J. (2014) Lipid organization of the plasma membrane. J. Am. Chem. Soc. *136*, 14554–14559.

Krieg, M., Fläschner, G., Alsteens, D., Gaub, B.M., Roos, W.H., Wuite, G.J.L., Gaub, H.E., Gerber, C., Dufrêne, Y. F., and Müller, D.J. (2019) Atomic force microscopy-based mechanobiology. Nat. Rev. Physics *1*, 41–57.

Lecordier, L., Walgraffe, D., Devaux, S., Poelvoorde, P., Pays, E., and Vanhamme, L. (2005) *Trypanosoma brucei* RNA interference in the mammalian host. Mol. Biochem. Parasitol. *140*, 127-131.

Lins, L., Thomas-Soumarmon, A., Pillot, T., Vandekerckhove, J., Rosseneu, M., and Brasseur, R. (1999) Molecular determinants of the interaction between the c-terminal domain of Alzheimer's β-amyloid peptide and apolipoprotein E α-helices, J. Neurochem. *73*, 758–769.

Marrink, S.J., Risselada, H.J., Yefimov, S., Tieleman, D.P., and de Vries, A.H. (2007) The MARTINI force field: coarse grained model for biomolecular simulations. J. Phys. Chem. B *111*, 7812–7824.

Meijering, E., Dzyubachyk, O., and Smal, I. (2012) Methods for cell and particle tracking. Methods in Enzymology *504*, 183-200.

Monticelli, L., Kandasamy, S.K., Periole, X., Larson, R.G., Tieleman, D.P., and Marrink, S. (2008) The MARTINI coarse-grained force field: extension to proteins. J. Chem. Theory Comput. *4*, 819–834.

Poon, S.K., Peacock, L., Gibson, W., Gull, K., and Kelly, S. (2012) A modular and optimized single marker system for generating *Trypanosoma brucei* cell lines expressing T7 RNA polymerase and the tetracycline repressor. Open Biol. *2*, 110037.

Schillers, H., Rianna, C., Schäpe, J., Luque, T., Doschke, H., Wälte, M., Uriarte, J.J., Campillo, N., Michanetzis, G.P.A., Bobrowska, J., et al. (2017) Standardized nanomechanical atomic force microscopy procedure (SNAP) for measuring soft and biological samples. Scient. Reports *7*, 5117.

Schmid, N., Eichenberger, A.P., Choutko, A., Riniker, S., Winger, M., Mark, A.E., and van Gunsteren, W.F. (2011**)** Definition and testing of the GROMOS force-field versions 54A7 and 54B7. Eur. Biophys. J. *40*, 843–856.

Schrödinger, L. (2010) The PyMOL molecular graphics system, version 1.3.

Wassenaar, T.A., Pluhackova, K., Böckmann, R.A., Marrink, S.J., and Tieleman, D.P. (2014**)** Going backward: a flexible geometric approach to reverse transformation from coarse grained to atomistic models. J. Chem. Theory Comput.*10*, 676-690.
